# Functional genomics elucidates regulatory mechanisms of Parkinson’s disease-associated variants

**DOI:** 10.1186/s12916-022-02264-w

**Published:** 2022-02-16

**Authors:** Rui Chen, Jiewei Liu, Shiwu Li, Xiaoyan Li, Yongxia Huo, Yong-Gang Yao, Xiao Xiao, Ming Li, Xiong-Jian Luo

**Affiliations:** 1 Key Laboratory of Animal Models and Human Disease Mechanisms of the Chinese Academy of Sciences & Yunnan Province, Kunming Institute of Zoology, Chinese Academy of Sciences, Kunming, 650223 Yunnan China; 2 Kunming College of Life Science, University of Chinese Academy of Sciences, Kunming, 650204 Yunnan China; 3 CAS Center for Excellence in Brain Science and Intelligence Technology, Chinese Academy of Sciences, Shanghai, China; 4 Zhongda Hospital, School of Life Sciences and Technology, Advanced Institute for Life and Health, Southeast University, Nanjing, 210096 Jiangsu China; 5 KIZ-CUHK Joint Laboratory of Bioresources and Molecular Research in Common Diseases, Kunming Institute of Zoology, Chinese Academy of Sciences, Kunming, 650223 Yunnan China; 6 Center for Excellence in Animal Evolution and Genetics, Chinese Academy of Sciences, Kunming, 650223 Yunnan China

**Keywords:** Parkinson’s disease (PD), Genome-wide association studies (GWASs), Single-nucleotide polymorphisms (SNPs), Functional genomics, Transcription factor (TF) binding, Regulatory mechanisms

## Abstract

**Background:**

Genome-wide association studies (GWASs) have identified multiple risk loci for Parkinson’s disease (PD). However, identifying the functional (or potential causal) variants in the reported risk loci and elucidating their roles in PD pathogenesis remain major challenges. To identify the potential causal (or functional) variants in the reported PD risk loci and to elucidate their regulatory mechanisms, we report a functional genomics study of PD.

**Methods:**

We first integrated chromatin immunoprecipitation sequencing (ChIP-Seq) (from neuronal cells and human brain tissues) data and GWAS-identified single-nucleotide polymorphisms (SNPs) in PD risk loci. We then conducted a series of experiments and analyses to validate the regulatory effects of these (i.e., functional) SNPs, including reporter gene assays, allele-specific expression (ASE), transcription factor (TF) knockdown, CRISPR-Cas9-mediated genome editing, and expression quantitative trait loci (eQTL) analysis.

**Results:**

We identified 44 SNPs (from 11 risk loci) affecting the binding of 12 TFs and we validated the regulatory effects of 15 TF binding-disrupting SNPs. In addition, we also identified the potential target genes regulated by these TF binding-disrupting SNPs through eQTL analysis. Finally, we showed that 4 eQTL genes of these TF binding-disrupting SNPs were dysregulated in PD cases compared with controls.

**Conclusion:**

Our study systematically reveals the gene regulatory mechanisms of PD risk variants (including widespread disruption of CTCF binding), generates the landscape of potential PD causal variants, and pinpoints promising candidate genes for further functional characterization and drug development.

**Supplementary Information:**

The online version contains supplementary material available at 10.1186/s12916-022-02264-w.

## Background

Parkinson’s disease (PD) is a leading neurodegenerative disease characterized by the presence of Lewy bodies and the loss of dopaminergic and other cells in the substantia nigra [1–5]. A core symptom of PD is the motor-related movement disorder, including rest tremor (or shaking), rigidity, impaired balance and coordination, bradykinesia, and difficulty with walking [1]. In addition to the classic motor-related symptoms, PD is also associated with nonmotor symptoms such as cognitive impairments, olfactory dysfunction , sleep disorders, and psychiatric symptoms [6]. PD prevalence increases dramatically with age and peaks at around 80 years old [1], and over 6 million people worldwide are affected by PD [7]. With the rise of life expectancy and the increase of aging population, the number of PD cases is estimated to grow by over 50% by 2030 [8].

So far, the mechanisms of dopaminergic cell loss in PD are not fully understood. However, accumulating evidence indicates that both genetic and environmental factors are involved in PD pathogenesis. Environmental factors, including exposure to pesticides [9], history of head injuries [10], rural residence, and the use of Beta-blockers [11], have been reported to be associated with the development of PD. Besides, the genetic heritability of PD is estimated to be around 22.7% [12], indicating an important role of genetic factors in this disease. Approximately 5–10% of PD cases are attributed to autosomal dominant or recessive inheritance [13], and several pathogenic genes such as *SNCA*, *LRRK2*, *PARK2*, and *PINK1* have been identified [12]. Nevertheless, mutations of these genes only explain a small proportion of PD cases, yet most PD cases develop a non-Mendelian form due to a combination of genetic and environmental factors. To identify risk variants for PD, several genome-wide association studies (GWAS) have been conducted and multiple risk loci have been identified [12, 14–18], providing some novel insights into the genetic architecture of PD. However, challenges remain in elucidating the genetic mechanisms of PD. First, the majority of the PD risk variants identified by GWAS are located in noncoding regions [19], suggesting that they might confer the risk of PD by regulating gene expression rather than directly changing the coding sequences of genes. This hypothesis is supported by a recent discovery that PD-associated variants are enriched in regulatory regions [19]. Second, identifying functional variations in the risk loci and elucidating their regulatory mechanisms remain difficult due to the complexity of linkage disequilibrium (LD) and gene regulation.

To address these challenges, we have herein systematically performed the first functional genomics study of PD. Through integrating chromatin immunoprecipitation sequencing (ChIP-Seq) and position weight matrix (PWM) data, we identified 44 TF binding-disrupting SNPs in 11 PD risk loci. We further validated the regulatory effects of 15 TF binding-disrupting SNPs with a series of experiments, including reporter gene assays, allele-specific expression (ASE), transcription factor (TF) knockdown, and CRISPR-Cas9-mediated genome editing. In addition, we also prioritized the potential target genes of these TF binding-disrupting SNPs using eQTL analysis. Finally, we compared the expression levels of the prioritized target genes in PD cases versus controls using expression data from a recent study by Marshall et al. [20]*.* Our study demonstrates the complex regulatory structure of PD risk variants (including widespread disruption of CTCF binding), identifies novel target genes regulated by the functional PD risk variants, and shows expression dysregulation of several target genes in PD cases. These results provide potential targets for the development of novel diagnostic and therapeutic strategies for PD.

## Methods

### GWASs used in this study

We used the genome-wide significant (GWS) SNPs reported by Nalls et al. [21] and Chang et al. [18]. In brief, Nalls et al. [21] performed a meta-analysis of PD GWAS (including 13,708 cases and 95,282 controls) and identified 27 GWS loci. Chang et al. [18] identified 41 GWS loci (17 novel) by meta-analyzing 26,035 PD cases and 403,190 controls. In total, 44 GWS index SNPs (Additional file 1: Table S1) [18, 21] from studies of Nalls et al. [21] and Chang et al. [18] were used in this study. Detailed information about the PD GWASs can be found in previous studies [18, 21].

### Extraction of SNPs in LD with the index SNPs

In order to capture potential common variants that are in LD with the 44 GWS index SNPs [18, 21], we extracted SNPs in LD (*r*^2^ > 0.6) with each index SNP using genotype data of Europeans (as most of PD risk variants were identified in populations of European ancestry) from the 1000 Genomes project [22]. Considering that different LD thresholds (*r*^2^) were used in different genetic studies to define whether interest SNPs were in LD, for example, Shriner et al. [23] and Chen et al. [24] used *r*^2^ ≥ 0.3 to select variants in LD with the reported risk variants, Ardlie et al. [25] showed that an *r*^2^ of 1/3 might be useful for LD determination for genetic mapping, Lee et al. [26] used *r*^2^ > 0.5 and the schizophrenia working group of the Psychiatric Genomics Consortium [27] used *r*^2^ > 0.6 to define whether flanking SNPs were in LD with the reported risk variants, we performed an extensive literature search to select a proper *r*^2^ threshold in this study. We noted that *r*^2^ > 0.6 was widely used to extract SNPs in high LD with the reported lead SNPs in many studies [28–41]. Of note, though *r*^2^ > 0.8 was used to define SNPs in strong LD [42–44], we utilized the widely accepted threshold (*r*^2^ > 0.6) in this study based on following considerations: First, *r*^2^ > 0.6 was widely accepted to define SNPs in high LD with the reported index SNPs [28–41]. Second, we considered both the degree of LD and the number of included SNPs. A more stringent *r*^2^ (e.g., 0.8) reduces the number of included SNPs, which may result in omission of many potential functional SNPs. Third, previous studies have showed that functional SNPs might be in low LD with the reported lead SNPs in some cases [45–47]. We thus selected the widely used *r*^2^ threshold (*r*^2^ > 0.6) in this study. PLINK software (version 1.9) [48] was used for LD analysis and SNP extraction. Genotype data of 503 Europeans from the 1000 Genomes project (Phase 3) were downloaded for LD calculation. We performed LD analysis to extract LD SNPs of the PD GWS index SNPs, and only SNPs located within 1 MB of the index SNPs were included (--ld-window-kb 1000). LD value (*r*^2^ cutoff) was set at 0.6 (--ld-window-r^2^ 0.6); thus, SNPs were extracted if the LD values between these SNPs and the index SNP exceeds 0.6.

### Functional genomics pipelines used to identify risk SNPs that affect TF binding

Our functional genomics pipelines include 3 major steps: Firstly, ChIP-Seq experiments performed in human brain tissues or neuronal-associated cell lines were obtained from ENCODE [49]. Secondly, we used MEME [50] to derive the DNA binding motifs of each TF, with the use of the obtained ChIP-Seq data from ENCODE. Thirdly, we extracted the flanking sequence of the SNPs that are in LD with the reported index SNPs. We then used FIMO [51] to scan whether the flanking sequence around each test SNP containing binding motif of TFs. Detailed procedures are as follows:

#### Step 1: ChIP-Seq data processing

To identify the DNA binding motifs of TFs, we downloaded the ChIP-Seq data from ENCODE (https://www.encodeproject.org/) [49]. The tissues/cell lines downloaded from ENCODE included astrocytes of the cerebellum, BE2C, brain microvascular endothelial cell, choroid plexus epithelial cell, H54, medulloblastoma, neural cell derived from H1-hESC, neural cell, PFSK-1, SH-SY5Y, SK-N-MC, and SK-N-SH. More details about the ChIP-Seq data have been described in our previous studies [52, 53]. As PD is a brain disorder, only ChIP-Seq data (a total of 34 TFs) from human brain tissues and neuronal cell lines were downloaded. Detailed processing pipelines have been described in previous studies [52–54]. Briefly, the downloaded Fastq files were firstly processed using the FastQC software (http://www.bioinformatics.babraham.ac.uk/projects/fastqc/) to evaluate sequence quality, and low-quality reads and adapter sequences were removed using Btrim64 (with the use of parameters “-a 20 -l 20”) [55]. Clean reads were then mapped to the human hg19 reference genome using Bowtie (version 1.1.2) (with the following parameters: “-n 2 -e 70 -m 2 -k 2”) [56]. The mapped SAM files were further converted into bam format, then sorted and indexed using samtools software [57]. Finally, MACS (version 1.4) software [58] was used for peak calling by using the converted bam files (with the use of parameters: “–keep-dup=1 -f BAM -w -S –call-subpeaks -g hs”). After quality control, ChIP-Seq data of 30 TFs were retained for further analysis.

#### Step 2: Motif discovery of TFs

To derive the binding motifs of each TF, we performed motif analysis using the MEME algorithm [50]. Briefly, TF ChIP-Seq peaks with FDR < 0.05 (compared with its corresponding negative control) and flanking sequences (± 20 bp) of the top 500 peaks (ranked by peak height) were extracted. The extracted sequences were then analyzed using MEME [50] to derive the binding motifs with the following parameters: “-nmotifs 5 -minw 6 -maxw 20.” Position weight matrix (PWM) is used to represent the binding sequence of a specific motif, and PWM could be used to represent consensus sequences (which reflect the pattern of a set of biological sequences) [50]. The derived motifs (from the ChIP-Seq data) were further compared with public TF motif databases, which include 7699 PWMs from JASPAR, Uniprobe, Hi-SELEX, and other resources (please refer to our previous papers for details [52, 53]), and the best-matched motif was used for further analysis.

#### Step 3: Identification of TF binding-disrupting SNPs

To test whether different alleles of the test SNPs affect TF binding, we firstly extracted flanking sequence (± 20 bp) of each test SNP. These sequences (41 bp, surrounding each test SNP), and the DNA binding motifs (derived from ChIP-Seq data, then compared with PWM databases to obtain the best-matched motifs) were used as inputs for find individual motif occurrences (FIMO) analysis [51]. To identify whether a given PWM occurred in the genomic sequence containing a given SNP, FIMO was used to scan the genomic sequence (containing a given SNP). The matched PWM overlaps with the test SNP for at least one base and the FIMO log-likelihood ratio (LLR) was set to *P* < 1 × 10^−3^ to define whether a SNP affected TF binding affinity.

**Fig. 1:**
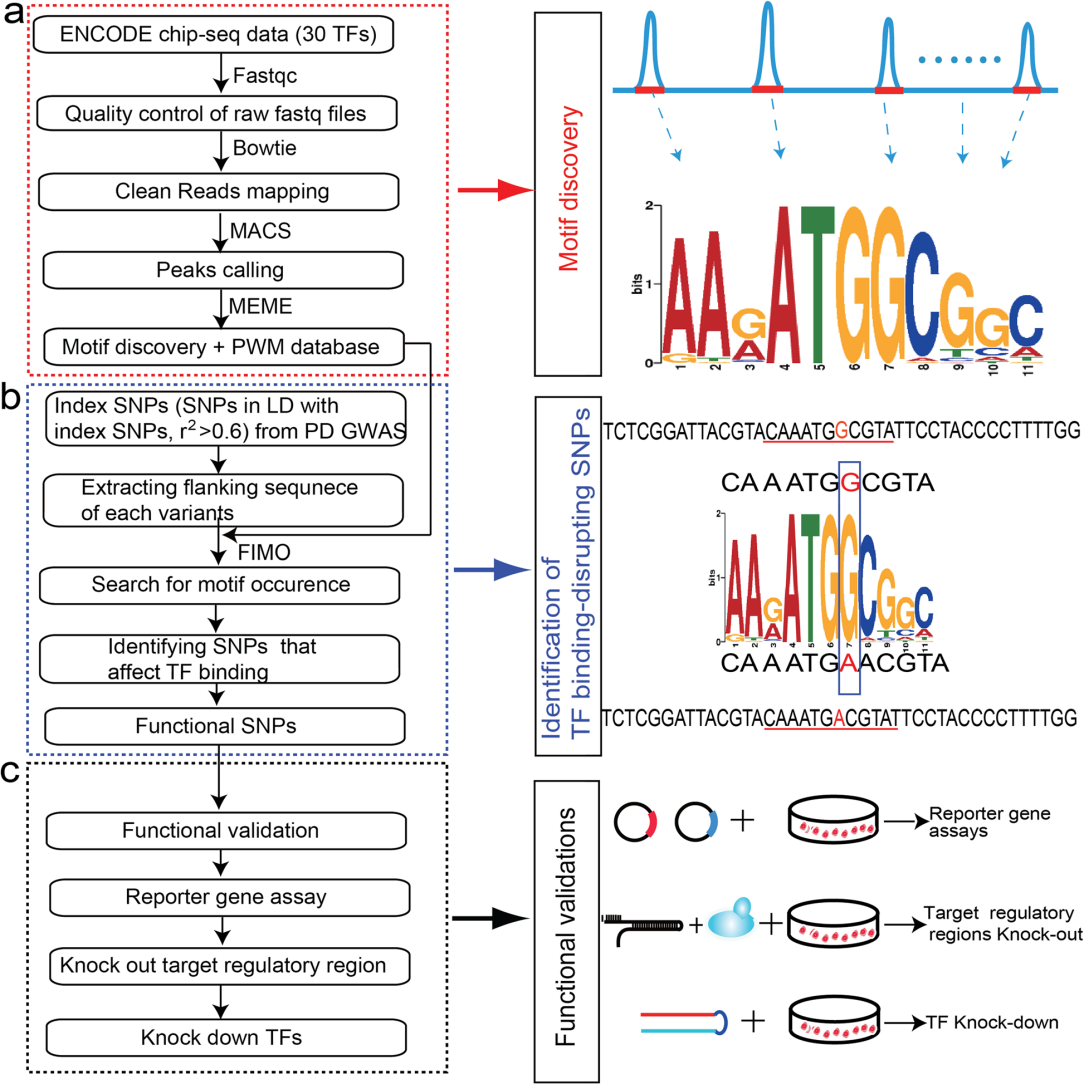
Overview of the study. **a** We first processed the ChIP-Seq data (from human brain or neuronal cells) to derive the binding motifs of the transcription factors (TFs). **b** We then investigated if the index SNPs (or SNPs in LD with the index SNPs) disrupt binding of TFs using find individual motif occurrences (FIMO). **c** Finally, a series of experiments and analyses were conducted to validate the regulatory effects of the TF binding-disrupting SNPs

In summary, we firstly used FastQC, Btrim64 [55], Bowtie [56], MACS [58] for quality control, reads mapping, and peak calling of the ChIP-Seq data. We then utilized MEME [50] to analyze the called peaks for each TF to obtain DNA binding motifs of TFs. Finally, we used FIMO [51] to scan motif occurrence around the flanking sequence of the test SNP. The flow chart of our functional genomics study is shown in Fig. 1, and more detailed information can be found in previous studies [52–54].

### Annotation of variants

To examine the genomic locations of the identified SNPs, we used ANNOVAR [59] (https://annovar.openbioinformatics.org/en/latest/) for variant annotation. The annotation files (hg19 genome build) were downloaded for annotation.

### DNase-Seq and histone modification analysis

We explored whether a given SNP was located in an actively transcribed genomic region by using DNase-Seq and histone modification data from the human brain tissues or neuronal-associated cell lines. Detailed information about DNase-Seq and histone modification analyses was described in our previous studies [52, 53].

### Cell culture

The SH-SY5Y (human neuroblastoma cell line) and U251 (human glioblastoma cell line) cell lines used in this study were obtained from the Cell Bank of Kunming Institute of Zoology, Chinese Academy of Sciences. SH-SY5Y cells were cultured in high-glucose DMEM (Gibco, Cat. No: C12430500BT) supplemented with 10% FBS (Gibco, Cat. No: 10091148), 10 mM sodium pyruvate solution (Gibco, Cat. No: 11360070), 1% penicillin and streptomycin (100 U/ml), and 1× minimum essential medium nonessential amino acid solution (Gibco, Cat. No: 11140050). U251 cells were cultured in high-glucose DMEM (Gibco, Cat. No: C11995500BT) supplemented with 10% FBS (Gibco, Cat. No: 10091148), and 1% penicillin and streptomycin (100 U/ml). Cells were passaged when the density reached about 80 to 90% confluence. Cells were cultured at 37 °C in 5% CO_2_. Mycoplasma test (PCR) was conducted periodically to make sure that these cell lines were mycoplasma-free.

### Vector construction

Based on the genomic locations of the test SNPs, we used pGL4.11[luc2P] vector and pGL3 promoter vector in this study. If the test SNPs were located in the promoter regions, the pGL4.11[luc2P] vectors were used. Otherwise, pGL3 promoter vectors were used. Specific primers (Additional file 1, Table S2) were used to amplify the genomic sequences (about 300–800 bp) containing the target SNPs. The obtained genomic sequences were then cloned into reporter vectors. After transforming DH5α cells, single colonies were selected and Sanger sequencing was used to confirm the sequences of inserted regions. More detailed information about vector construction can be found in our previous studies [52, 53].

### Reporter gene assays

SH-SY5Y and U251 cells were transfected with the constructed pGL3 promoter or pGL4.11[luc2P] vectors. The pRL-TK Renilla vector was used as the internal control. SH-SY5Y and U251 cells were plated into 96-well plates at densities of 1.0 × 10^5^ cells/well and 1.0 × 10^4^ cells/well, respectively. After culture for 12 h, Lipofectamine™ 3000 (Invitrogen, Cat.No: L3000-015) was used to transfect the above vectors. SH-SY5Y and U251 cells were transfected with 150 ng of the pGL4.11[luc2P] or the pGL3, and 50 ng of the pRL-TK Renilla as the internal control. Forty-eight hours posttransfection, luciferase activity was measured by a dual luciferase reporter gene assay system (Promega, Cat.No: E1960). Differences were calculated with two-tailed Student’s *t* test, and the significance threshold was set at *P* < 0.05.

### Allele-specific expression analysis

The imbalanced expression of the two parental alleles is called allele-specific expression (ASE). ASE analysis is a within-individual analysis that compares the expression levels of a specific transcript with different alleles on a specific SNP using RNA sequencing (RNA-Seq) data. ASE analysis requires that the test SNP in the transcript is heterozygous. The expression level of a specific transcript in an individual is quantified by RNA-Seq, and if this transcript contains a heterozygous SNP of interest, the counts of this transcript containing either reference allele or alternative allele were calculated. The transcript counts ratio between the two alleles was compared with the expected null ratio by a Binomial test to determine the significance of ASE of a variant. We utilized Genotype-Tissue Expression Version 8 (GTEx V8) data (only brain tissues were included) to explore whether the 44 TF binding-disrupting SNPs identified in this study showed ASE in the human brain tissues [60, 61]. The GTEx Consortium (V8) performed ASE analysis as follows. Firstly, the GTEx RNA-seq data were mapped to hg38 reference genome by STAR software [62]. Secondly, the SNP-level ASE were detected by GATK ASEReadCounter tool [63], which requires RNA-Seq bam files (per subject across all tissues) and vcf files (contains the genotype of the variants) to perform ASE. Thirdly, for each SNP in the raw ASE output, only SNPs with ≥ 8 reads were retained. For each SNP, the expected null ratio is calculated, and a Binomial *P* is used to determine the statistical significance of the ASE (by comparing the ratio of RNA-Seq ref/alt allele with the expected null ratio). More details about ASE analysis can be found in GTEx original papers (https://gtexportal.org/) [60, 61].

### eQTL analysis

The brain eQTL data sets used in this study were from four previous studies: the Common Mind Consortium (CMC) (*N* = 467) [64], the Genotype-Tissue Expression (GTEx) v7 (13 brain regions, N ranges from 80 to 154) [65], the Lieber Institute for Brain Development (LIBD) brain eQTL (*N* = 412) [66], and the xQTL map of the human brains (xQTL) (*N* = 494) [67]. Gene expression levels in all eQTL datasets were quantified with RNA-Seq. In brief, the CMC eQTL summary statistics were derived from the dorsolateral prefrontal cortex (DLPFC) of 467 subjects [64]. The GTEx dataset collected a total of 13 brain tissues from healthy subjects, with sample sizes ranging from 80 to 154 in different brain regions [65]. The LIBD dataset contains five levels (including gene, exon, junction, transcript, and expressed region) expression data from the DLPFC of 412 subjects [66], and only gene-level eQTLs were used in our study. The xQTL is a multi-omic dataset comprising RNA sequence, DNA methylation, and histone acetylation from the DLPFC of 494 individuals [67]. More detailed information can be found in previous studies [64–67].

### Knockdown of the corresponding TFs

We used short hairpin RNAs (shRNAs) to knock down the TFs and knockdown efficiency was assessed with real-time quantitative PCR (RT-qPCR). The following TFs were knocked down with shRNAs, including SIN3A, SMC3, CTCF, RAD21, and REST. The annealed shRNAs were ligated into the pLKO.1 vector, and the constructed vectors were used to transform Stbl3 competent cells (Beyotime, Cat.No: D0378) (produced using the Supercompetent Cell Preparation Kit (Beyotime, Cat.No: D0302)). DNA sequencing was used to verify the sequences of the inserted shRNAs. Lentiviral packaging vectors pMD2.G (Addgene, Cat. No: 12259) and psPAX2 (Addgene, Cat. No: 12260) and shRNA-expressing vector were cotransfected into HEK293T cells using the PEI transfection reagent (Sigma, Cat. No: 408727). Forty-eight hours posttransfection, the viral supernatants were harvested, filtered, and directly added into the culture medium of SH-SY5Y cells. The cells were selected with puromycin (2μg/mL) (Sigma, Cat. No: 540222) for 1 week. The shRNA sequences are provided in Additional file 1, Table S3.

### Knockout of genomic regions containing the target SNPs

To evaluate the potential regulatory impact of the genomic regions (containing the TF binding-disrupting SNP) on target genes, we used CRISPR-Cas9-mediated gene editing to knock out the given genome regions. For each genomic region of interest, two guide RNAs (sgRNAs) were designed with the CRISPR sgRNA Design Tool (https://zlab.bio/guidedesign-resources). PX459M and EZ-GuideXH were used to construct the knockout (KO) vector backbone. The vector PX459M and EZ-GuideXH were firstly linearized with the restriction enzyme BbsI, then expression constructs of the sgRNAs (sgRNA1 and sgRNA2)were prepared by cloning annealed sgRNAs into linearized PX459M and EZ-GuideXH vectors. After validating with Sanger sequencing, the construct expressing sgRNA2 from EZ-GuideXH was cloned into a linearized PX459M which express sgRNA1 with the restriction enzymes XhoI and HindIII. All recombinant plasmids were generated using the ClonExpress II One Step Cloning Kit (Vazyme, Cat.No: C112-01). And the knockout experiments were performed in HEK293T cells.

### Real-time quantitative PCR (RT-qPCR) analysis

Total RNA was extracted with TRIzol™ LS Reagent (Invitrogen, Cat.No: 10296028), treated with gDNA Eraser (Takara, Cat.No: RR047A) to remove potential genomic DNA and reversely transcribed into cDNA with PrimeScript™ RT Kit according to the manufacturer’s instructions. The expression levels of the target genes were determined by qPCR using TB Green™ Premix Ex Taq™ II (TliRNaseHPlus) (Takara, Cat.No: RR820A) in a QuantStudio™ 12K Flex (Applied Biosystems) instrument or a CFX96 Touch™ Real-Time PCR detection system. All of the experiments were conducted in triplicates, and gene expression was determined with the 2^−ΔΔCt^ method (ACTB was used as internal control) [68]. Primer sequences are provided in Additional file 1, Table S4. Differences were calculated with two-tailed Student’s *t* test, and the significance threshold was set at *P* < 0.05.

### Expression analysis of target genes in PD cases and controls

To explore the expression levels of the potential target genes of the identified TF binding-disrupting SNPs in PD cases and controls, we used the expression data generated by Marshall et al. [20]. Briefly, the prefrontal cortex of 24 PD cases and 12 controls were collected by Marshall et al. [20], and gene expression levels were quantified with RNA sequencing. Detailed information on sample description, tissue collection, RNA sequencing, and statistical analyses were provided in the original paper [20].

### Brain single-cell expression analysis

We used the Cortical Development Expression (CoDex) viewer to perform single-cell expression analysis of the PD target genes identified in this study [69]. CoDex viewer includes 40,000 single-cell RNA-Seq expression profiles from the developing human cortex. CoDEx is a user-friendly data portal that facilitates data access and browsing. The detailed information on sample collection information, data processing, cell clustering, and analysis approaches have been described in the original paper [69] and the CoDex viewer website (http://solo.bmap.ucla.edu/shiny/webapp/).

## Results

### Functional genomics identified 44 TF binding-disrupting PD risk SNPs

**Fig. 2:**
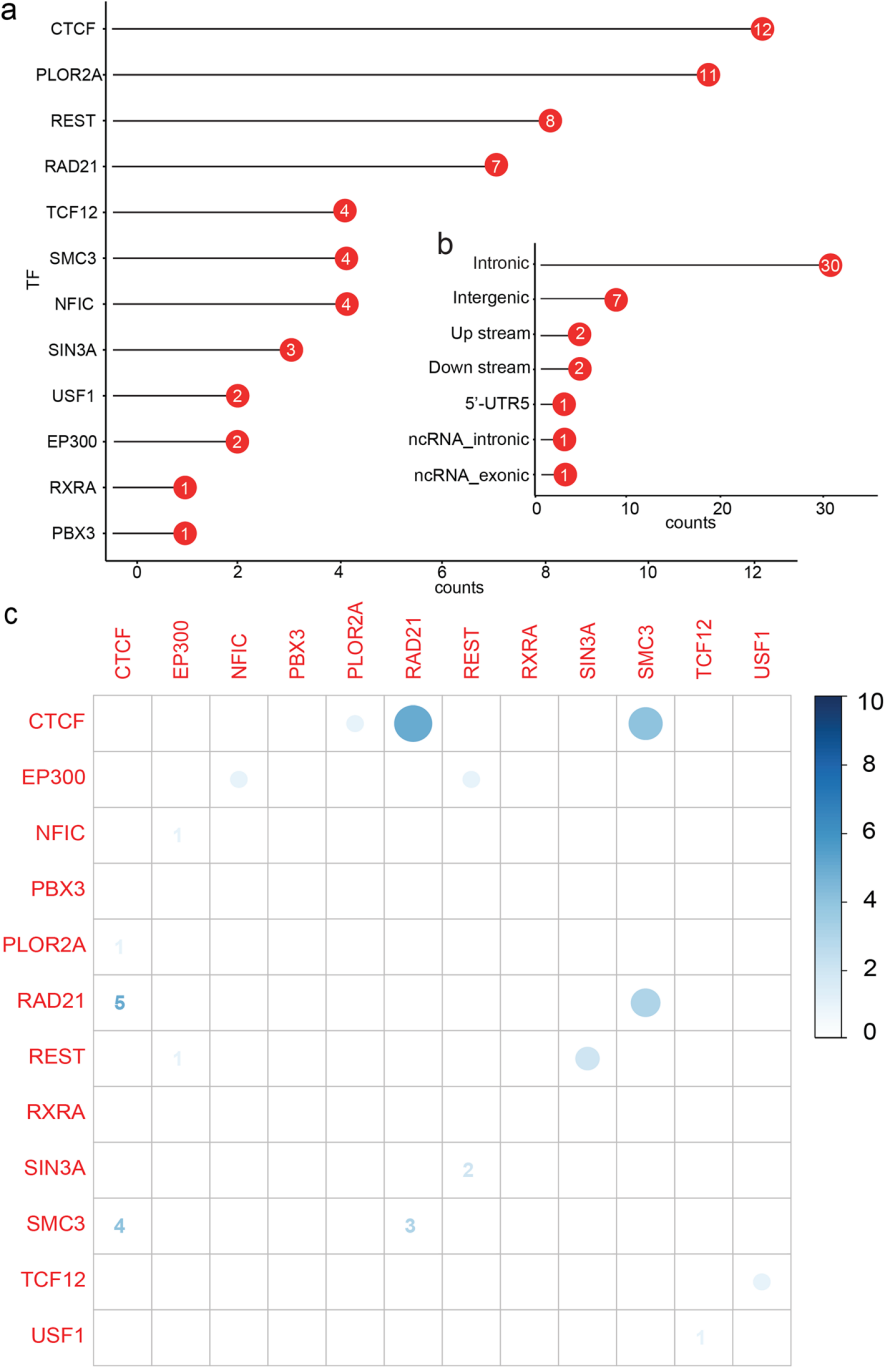
The distribution of TF binding-disrupting SNPs in the human genome. **a** The number of SNPs that affect the binding affinity of each TF. **b** Distribution of the binding-disrupting SNPs in the human genome. **c** Heatmap showed the number of SNPs that affect binding of two or more TFs

We first extracted the SNPs in LD (*r*^2^ > 0.6) with the 44 index SNPs reported by two PD GWASs [18, 21]. In total, 6288 SNPs were extracted (Additional file 2, Table S5). By integrating the index SNPs (including SNPs in LD with the index SNPs) and the DNA binding motifs derived from ChIP-Seq data (Fig. 1), we identified 44 SNPs that disrupted the binding of 12 TFs (Fig. 2 and Additional file 1, Table S6). Among the 44 TF binding-disrupting SNPs, 12 disrupted CTCF binding, 11 disrupted POLR2A binding, 8 disrupted REST binding, and 7 disrupted RAD21 binding (Fig. 2a). These 44 TF binding-disrupting SNPs were from 11 PD risk loci (Additional file 1, Table S6). Of note, approximately 84% (37/44) TF binding-disrupting SNPs were located in the intronic and intergenic regions (Fig. 2b), suggesting their potential regulatory impact on transcription. We noticed that a small proportion of SNPs disrupted binding of two or three TFs (Fig. 2c), e.g., 5 SNPs disrupted the binding of CTCF and RAD21, and 4 SNPs disrupted the binding of CTCF and SMC3. These results identified the functional (or potential causal) SNPs in the reported PD risk loci, indicating that they may confer risk for PD through affecting TF binding. In addition, these results also suggested that the TF binding-disrupting SNPs may represent the potential causal variants at these risk loci.

### Reporter gene assays validated the regulatory effects of 15 identified TF binding-disrupting SNPs

**Fig. 3:**
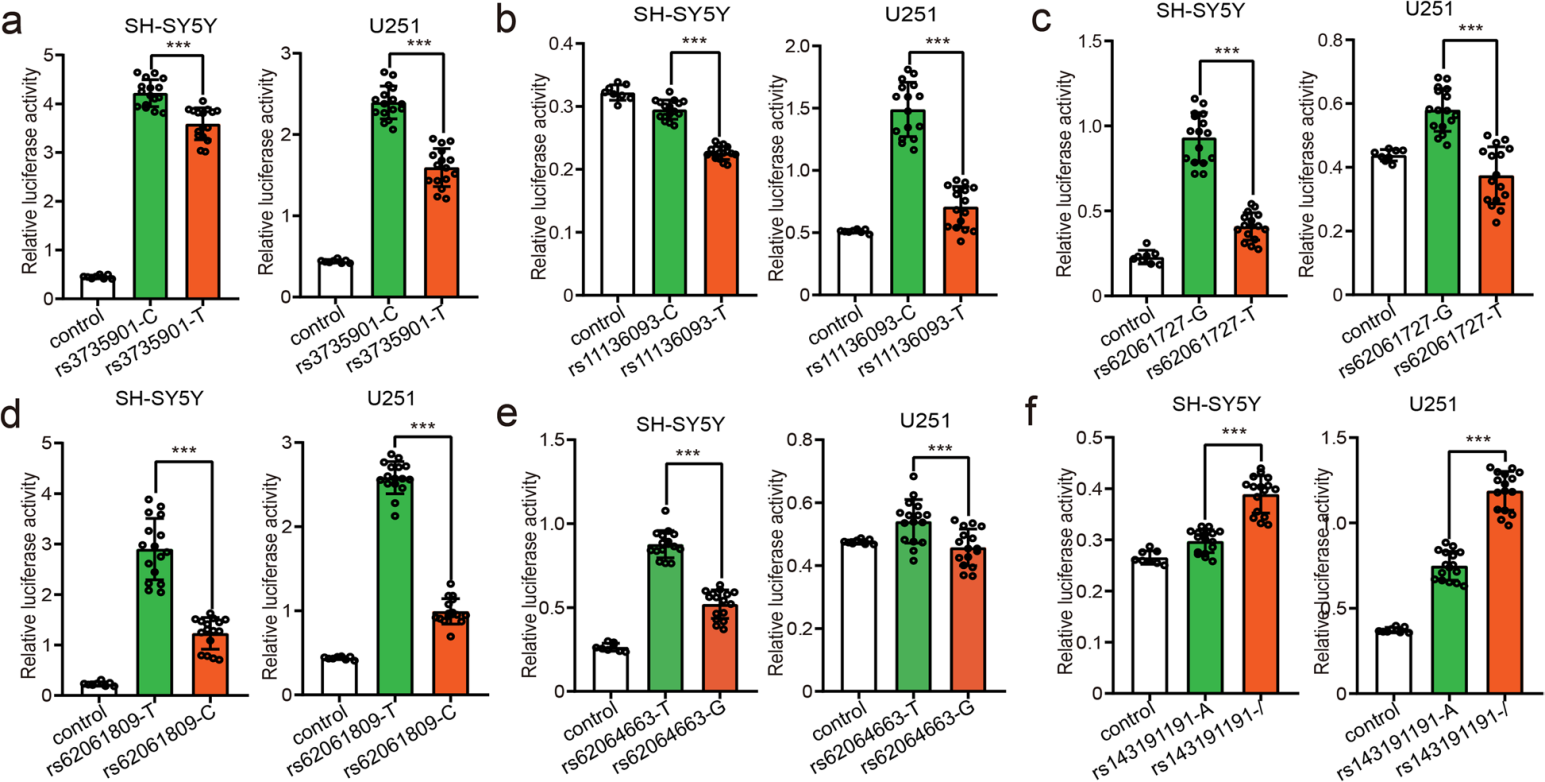
Validation of the regulatory effects of the TF binding-disrupting SNPs with dual luciferase reporter gene assays. Six TF binding-disrupting SNPs showed significant differences between different alleles, with the same allelic effect direction in both SH-SY5Y and U251 cells. **a** The constructs containing the C allele of rs3735901 exhibited significantly higher luciferase activity than the construct containing the T allele in SH-SY5Y and U251 cells. **b** The C allele of rs11136093 conferred significantly higher luciferase activity than the T allele in SH-SY5Y and U251 cells. **c** The reporter vectors containing the G allele of rs62061727 displayed significantly higher luciferase activity than the T allele in SH-SY5Y and U251 cells. **d** The constructs carrying the T allele of rs62061809 exhibited significant higher luciferase activity than the constructs carrying the C allele in SH-SY5Y and U251 cells. **e** The reporter vectors containing the T allele of rs62064663 showed significantly higher luciferase activity than the G allele in SH-SY5Y and U251 cells*.* **f** The reporter vectors containing the single base deletion of rs143191191 showed significantly higher luciferase activity than the A allele in SH-SY5Y and U251 cells*. N* = 8 for the control group, *n* = 16 per experimental group for SH-SY5Y and U251 cells*.* Two-tailed Student’s *t* test was used for statistical analyses. *P < 0.05, **P < 0.01, ***P < 0.001

**Fig. 4:**
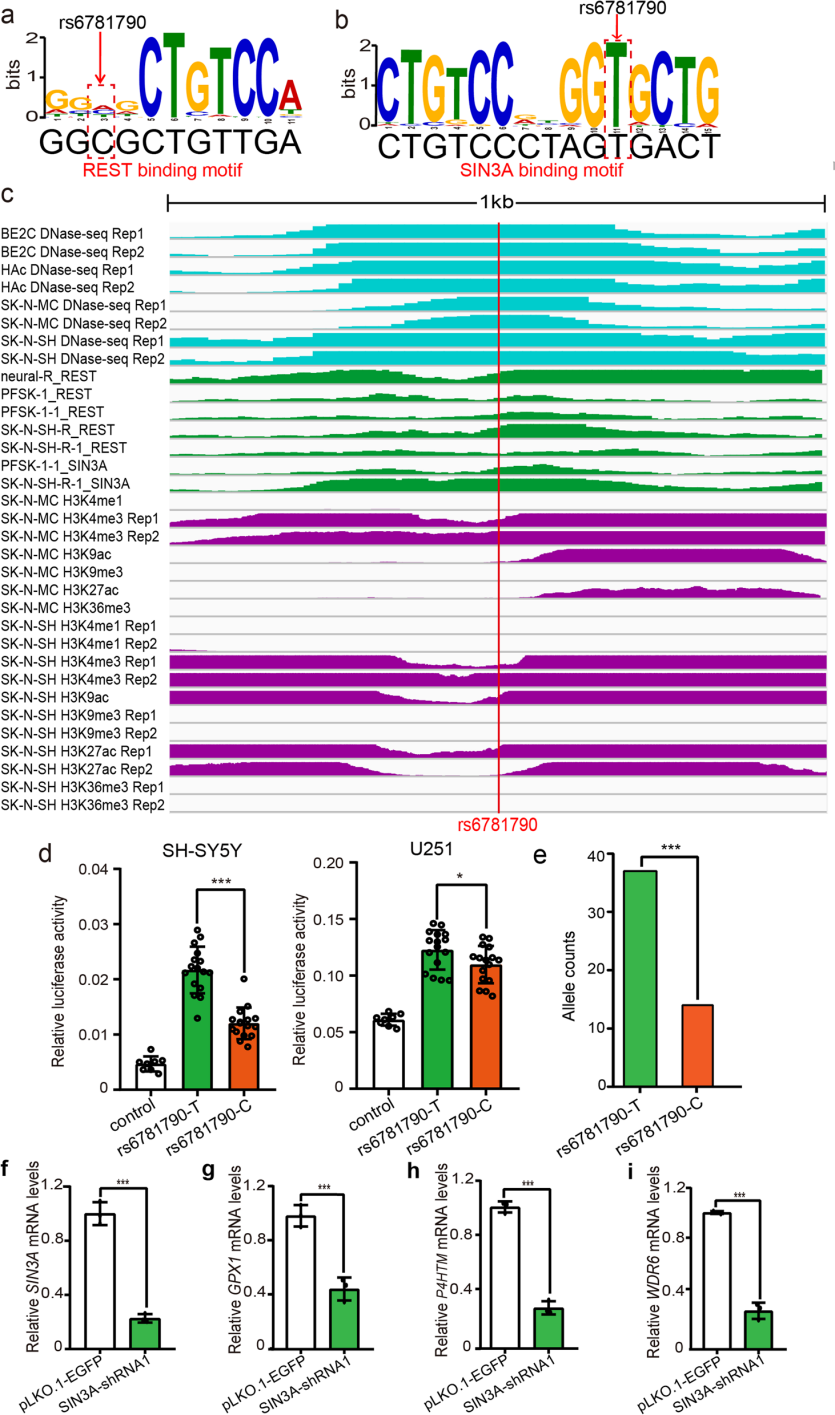
Disruption of REST and SIN3A binding by SNP rs6781790. **a,b** rs6781790 disrupts the binding of REST and SIN3A TFs. **c** The 1 kb genomic sequence surrounding SNP rs6781790 was displayed with DNase-Seq signal (light blue), the transcription factor (TF) chromatin immunoprecipitation, and sequencing (ChIP-Seq) signal (green), and histone modifications (purple). **d** Reporter gene assays showed that the T allele of rs6781790 conferred significantly higher luciferase activity than the C allele in SH-SY5Y and U251 cells. **e** Allele-specific expression (ASE) analysis showed that different alleles of rs6781790 exhibited significant preferential expression in human brain tissues. **f–i** SIN3A knockdown resulted in significant downregulation of *GPX1*, *P4HTM*, and *WDR6*, indicating that these genes are regulated by the SIN3A. *N* = 8 for the control group, *n* = 16 per experimental group in SH-SY5Y and U251 cells. *n* = 3 per group in **f–i**. Two-tailed Student’s *t* test was used for statistical analyses. **P < 0.01, ***P < 0.001

**Fig. 5:**
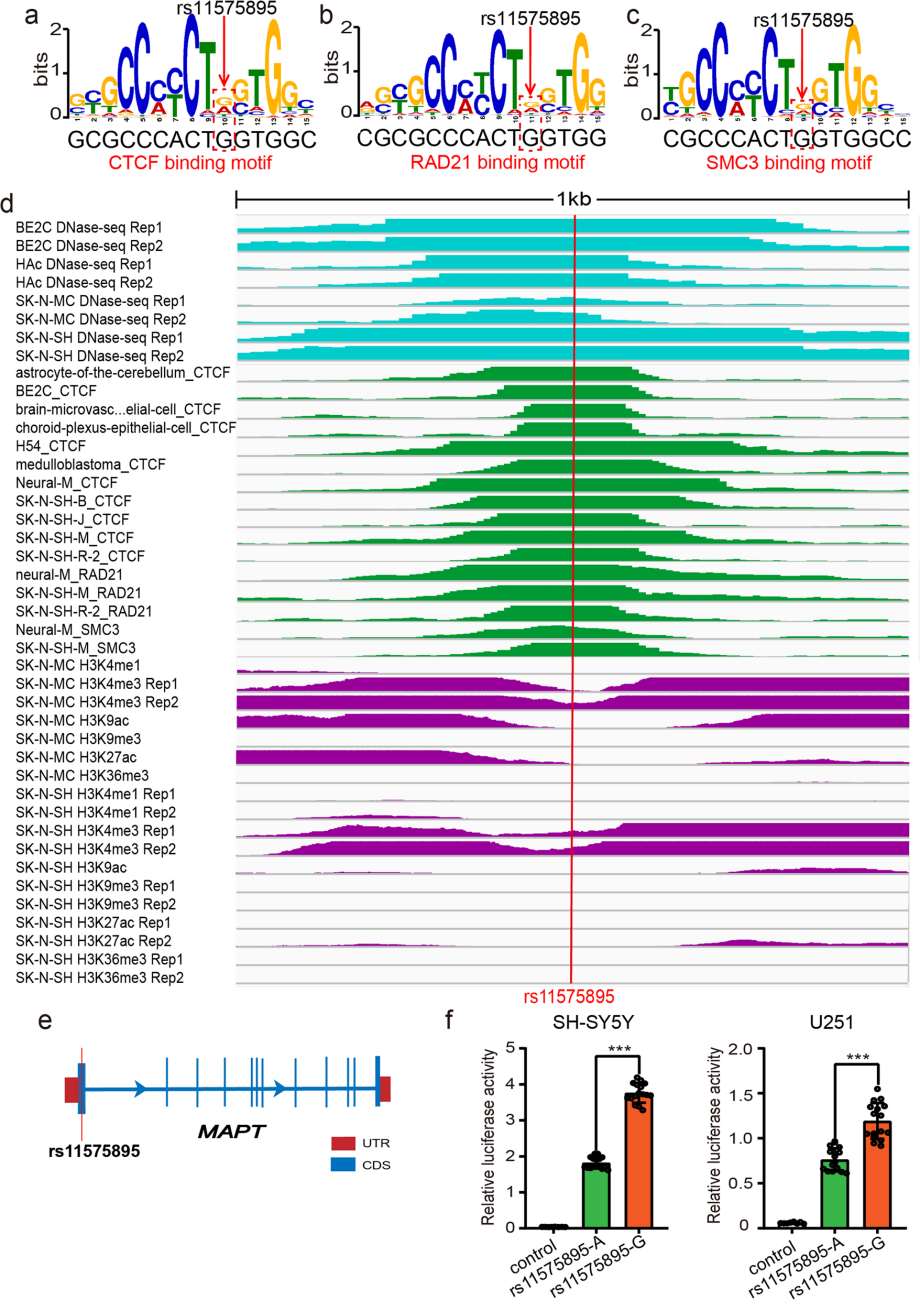
Disruption of CTCF, RAD21, and SMC3 binding by SNP rs11575895. **a–c** Disruption of CTCF, RAD21, and SMC3 binding by SNP rs11575895. **d** SNP rs11575895 is located in a genomic region with strongly DNase-Seq, ChIP-Seq, and histone modification signals, indicating that rs11575895 is located in a region of active transcription in neuronal cells. **e** SNP rs11575895 is located in the first exon of the longest transcript of *MAPT*. **f** Reporter gene assays exhibited that the G allele of rs11575895 conferred significantly higher luciferase activity than the A allele in SH-SY5Y and U251 cells. *N* = 8 for the control group, *n* = 16 per experimental group for SH-SY5Y and U251 cells. Two-tailed Student’s *t* test was used for statistical analyses. **P < 0.01, ***P < 0.001

**Fig. 6:**
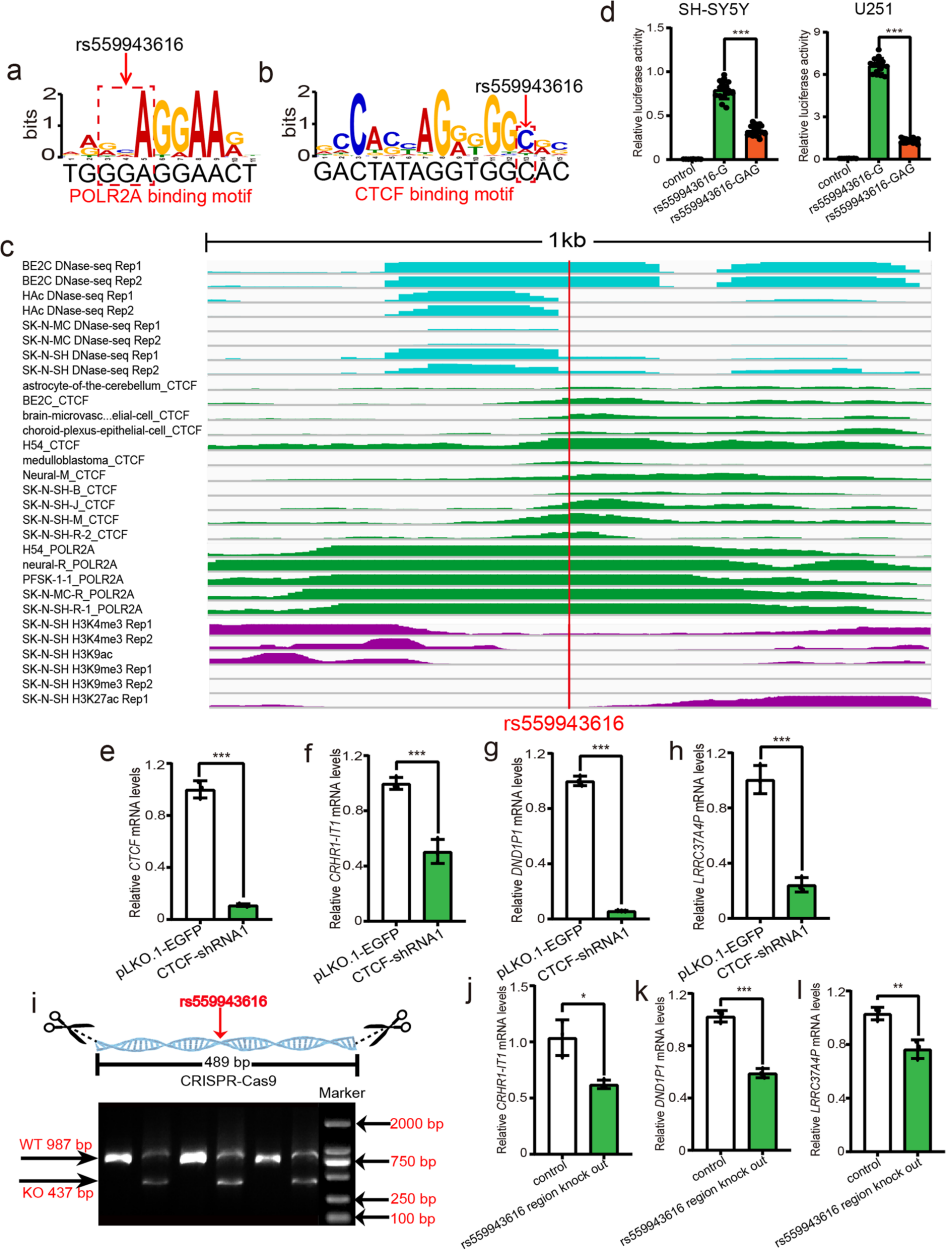
Verification of the regulatory effect of rs559943616 by reporter gene assays and CRISPR-Cas9-mediated genome editing. **a,b** SNP rs559943616 disrupts POLR2A and CTCF binding. **c** The 1 kb sequence surrounding SNP rs559943616 is marked with DNase-Seq, ChIP-Seq, and histone modification signals, indicating that rs559943616 is located in an actively transcribed genomic region in neuronal cells. **d** Reporter gene assays validated the regulatory effect of rs559943616. The G allele of rs559943616 conferred significantly higher luciferase activity than the GGA allele in SH-SY5Y and U251 cells. **e–h** CTCF knockdown resulted in significant downregulation of *CRHR1-IT1*, *DND1P1*, and *LRRC37A4P*, indicating that these genes are regulated by the CTCF. **i–l** CRISPR-Cas9-mediated genome editing revealed that deletion of the genomic region containing rs559943616 led to significant expression changes of *LRRC37A4P*, *DND1P1*, *CRHR1-IT1*. **i** Electrophoresis showed that the given genomic region containing rs559943616 was deleted. WT indicates that the length of the DNA fragments containing rs559943616 is 987 bp in wild-type cells. KO indicates that the length of the DNA fragments containing rs559943616 is 437 bp in edited cells. *N* = 8 for the control group, *n* = 16 per experimental group for SH-SY5Y and U251 cells, *n* = 3 per group in **e–h**, **j–l**. Two-tailed Student’s *t* test was used for statistical analyses. *P < 0.05, **P < 0.01, ***P < 0.001

Our functional genomic study identified 44 SNPs that disrupted the binding of 12 TFs. To further verify the regulatory effect of these TF binding-disrupting SNPs, we randomly selected 15 SNPs for reporter gene assays (Additional file 1, Table S7). Among the 15 tested SNPs, 11 (over 73%) showed regulatory effects (i.e., different alleles at these 11 SNPs affected the reporter gene activity significantly (uncorrected *P* < 0.05)) in both SH-SY5Y and U251 cells (Fig. 3, Additional file 1: Figure S1, Table S8). Of note, 9 TF binding-disrupting SNPs showed significant luciferase differences between two different alleles, with the same allelic effect direction in both SH-SY5Y and U251 cells (Figs. 3, 4, 5, and 6 and Additional file 1, Table S8), strongly suggesting the functionality of these 9 SNPs. Among these 9 SNPs, reporter gene assays of rs6781790 (Fig. 4), rs11575895 (Fig. 5), and rs559943616 (Fig. 6) are shown in Figs. 4, 5 and 6, and the reporter gene assays results of the remaining 6 SNPs are shown in Fig. 3. Four SNPs (rs7599054, rs117629202, rs145273500, and rs16833689) did not show regulatory effect in any of the cell lines. Collectively, the results provided robust evidence that the identified TF binding-disrupting SNPs were functional.

### ASE analysis supported the functionality of the identified TF binding-disrupting SNPs

To further investigate the regulatory effect of the identified TF binding-disrupting SNPs, we used the ASE data from GTEx (only brain tissues were used). We found that 13 out of 44 TF binding-disrupting SNPs showed ASE (Additional file 2, Table S9) in the human brain. That is, the expression level of the transcript (counts from RNA-Seq) containing the maternal allele was significantly different from the transcript containing the paternal allele, indicating that one allele was preferentially expressed compared with the other. In addition, we also found that 8 (rs6781790, rs10270788, rs2272718, rs878051, rs62064663, rs12150515, rs1468240, and rs17665188) out of the 13 ASE SNPs were in very high LD (*r*^2^ > 0.8) with coding SNPs (Additional file 2, Table S10), suggesting that these ASE SNPs may modify the penetrance of coding variants [70]. These ASE analyses further supported that the identified TF binding-disrupting SNPs were functional.

### Disruption of REST and SIN3A binding by rs6781790

We identified a TF binding-disrupting SNP (rs6781790) at 3p21.31. FIMO analysis showed that rs6781790 disrupted the binding of REST and SIN3A (Fig. 4a,b). ChIP-Seq data showed that REST and SIN3A can bind to the genomic region containing rs6781790 in the human brain tissues or neuronal cells (Fig. 4c). Consistent with ChIP-Seq data, DNase-Seq data revealed that rs6781790 is located in a genomic region with active transcription in brain tissues or neuronal cells (Fig. 4c). The histone modification data further confirmed that rs6781790 is located in an actively transcribed genomic region (i.e., active regulatory element) (Fig. 4c). We tested the regulatory effect of rs6781790 with reporter gene assays and found that the T allele of rs6781790 was associated with higher luciferase activity compared with the C allele in both SH-SY5Y cells and U251 cells, with the same direction of allelic effect (Fig. 4d). Finally, ASE analysis showed that the T allele was preferentially expressed compared with C allele (i.e., the counts of the transcript containing the T allele was significantly higher than the transcript containing the C allele, binominal test *P* = 6.37 × 10^−4^) (Fig. 4e). As rs6781790 disrupted SIN3A binding, we further investigated whether SIN3A knockdown (using shRNA) modulates the expression of potential target genes (i.e., eQTL genes) of rs6781790 in SH-SY5Y cells. We found that SIN3A knockdown resulted in significant downregulation of *GPX1*, *P4HTM*, and *WDR6* (eQTL genes of rs6781790) (Fig. 4f–i), indicating that SIN3A facilitated the regulatory effect of rs6781790 on these genes. Taken together, these consistent and convergent results indicated that rs6781790 is a regulatory SNP with functional consequences.

### Disruption of CTCF, RAD21, and SMC3 binding by rs11575895

Our functional genomics study identified rs11575895 as a TF binding-disrupting SNP at 17q21.31 (Fig. 5). rs11575895 affects the binding of CTCF, RAD21, and SMC3 (Fig. 5a–c). ChIP-Seq data demonstrated that CTCF, RAD21, and SMC3 could bind to the genomic sequence containing rs11575895 (Fig. 5d). The DNase-Seq and histone modification data also showed that rs11575895 is located in an active regulatory element (in the human brain tissues or neuronal cells) (Fig. 5d). Of note, we noticed that rs11575895 is located in the promoter region (or in the first exon, as *MAPT* has several transcripts with different lengths) of *MAPT* (Fig. 5e), a gene that was reported to be associated with PD in previous studies [14–16, 71–74].

Reporter gene assays showed that the vector containing G allele of rs11575895 exhibited significantly higher luciferase activity compared with A allele in both SH-SY5Y and U251 cells (Fig. 5f). Finally, as rs11575895 disrupted the binding of CTCF, RAD21, and SMC3 TFs, we explored whether the eQTL genes of rs11575895 were regulated by these TFs. Knockdown of CTCF resulted in significant downregulation of *CRHR1-IT1*, *DND1P1*, *LRRC37A4P*, and *MAPT* expression (Additional file 1, Figure S2). In contrast, knockdown of RAD21 led to significant upregulation of *CRHR1-IT1*, *DND1P1*, *LRRC37A4P*, and *MAPT* expression (Additional file 1, Figure S2). Interestingly, SMC3 knockdown resulted in increased expression of *CRHR1-IT1*, *DND1P1*, and *LRRC37A4P* and decreased expression of *MAPT* (Additional file 1, Figure S2). These results indicated that CTCF, RAD21, and SMC3 can regulate the expression of eQTL genes of rs11575895, and this process was likely mediated by the interaction between rs11575895 and these three TFs. These data demonstrated that rs11575895 is a functional variant with a regulatory effect.

### Disruption of POLR2A and CTCF binding by rs559943616

In addition to the abovementioned SNPs, we also found that rs559943616 disrupted the binding of POLR2A and CTCF (Fig. 6a,b). ChIP-Seq data showed that TFs POLR2A and CTCF could bind to the genomic sequence containing rs559943616, and DNase-Seq data showed that the genomic region containing rs559943616 is actively transcribed in human brain tissues or neuronal cells (Fig. 6c). We further verified the regulatory effect of rs559943616 with reporter gene assays and found that the vector containing the G allele exhibited significantly higher luciferase activity compared with that containing GGA allele in both SH-SY5Y and U251 cells (Fig. 6d). We further knocked down CTCF and found significant downregulation of *CRHR1-IT1*, *DND1P1*, and *LRRC37A4P* expression in CTCF knocked down cells (Fig. 6e–h), indicating that the expression of *CRHR1-IT1*, *DND1P1*, and *LRRC37A4P* were regulated by CTCF. Finally, CRISPR-Cas9-mediated genomic sequence deletion (489 bp) revealed that the genomic region containing rs559943616 can regulate the expression of *LRRC37A4P*, *DND1P1*, and *CRHR1-IT1* (Fig. 6i–l). Of note, we noticed that the expression of these three genes were downregulated in rs559943616 knocked-out cells compared with wild-type cells, suggesting that the genomic region containing rs559943616 may act as an enhancer for these three genes.

### eQTL analysis identified the potential target genes regulated by these TF binding-disrupting SNPs

We validated the regulatory effect of 15 identified TF binding-disrupting SNPs using a series of experiments, including reporter gene assays, TF knockdown, ASE analysis, and CRISPR-Cas9-mediated genome editing. These results suggested that the majority of TF binding-disrupting SNPs may exert their biological effect by regulating gene expression. We thus examined the associations between these SNPs and gene expression using four human brain eQTL datasets. Among the 44 TF binding-disrupting SNPs, 38 showed associations with gene expression (uncorrected, *P* < 0.05) in at least one eQTL dataset (Additional file 2, Table S11). Besides, 34 SNPs showed significant associations with gene expression in at least two brain eQTL datasets (Additional file 2, Table S12), and 19 SNPs showed significant associations with gene expression in at least three brain eQTL datasets (Additional file 2, Table S13). Of note, 12 SNPs showed significant associations with gene expression in all four brain eQTL datasets (Table 1), strongly suggesting the regulatory effect of these SNPs on gene expression. The boxplots of the eQTL analyses are provided in Additional file 1, Figure S3 [64, 66]. Collectively, our eQTL analyses linked the TF binding-disrupting SNPs to their potential target genes.

### Dysregulation of the potential target genes of the TF binding-disrupting SNPs in PD cases

We further explored the expression levels of the potential target genes of the TF binding-disrupting SNPs in the brains of PD cases and controls using the data from Marshall et al. [20]. Among the 103 eQTL genes of the TF binding-disrupting SNPs, four (*AMT*, *DALRD3*, *GPNMB*, and *RHOBTB2*) showed significantly varied mRNA levels (corrected, *q* < 0.05) in brains of PD cases compared with controls (Additional file 1, Table S14) [20], suggesting that these TF binding-disrupting SNPs may confer PD risk through regulating these genes.

## Discussion

Genetic studies, especially recent large-scale GWASs, have identified multiple PD risk loci showing robust associations with PD. Despite that these studies have provided important insights into the genetic etiology of PD, the potential causal variants in most loci and their roles in PD pathogenesis remain elusive. Extensive LD, the complexity of gene regulation, and the high degree of tissue specificity of most regulatory elements impede the identification of causal variants and the dissection of their pathogenic mechanisms. To identify the potential causal (or functional) variants in the reported PD risk loci and to elucidate their regulatory mechanisms, we have herein carried out a functional genomic study. We identified 44 SNPs (from 11 risk loci) affecting the binding of 12 TFs and we performed a series of experiments and analyses to validate their regulatory effects. In addition, we also identified the potential target genes regulated by these TF binding-disrupting SNPs through eQTL analysis. Finally, we showed that 4 eQTL genes of these TF binding-disrupting SNPs were dysregulated in PD cases compared with controls.

Our study provides novel insights into the genetic mechanisms of PD. First, we showed that the regulatory mechanisms of PD risk variants are complex. The 44 TF binding-disrupting SNPs disrupt the binding of 12 TFs, with approximately 27% (12/44) disrupting CTCF binding. Second, we identified the TF binding-disrupting SNPs from approximately 25% reported PD risk loci (11, a total of 44 GWS index SNPs were included in this study). These SNPs may represent promising functional or causal variants for these loci. Third, over 68% (30/44) of the 44 TF binding-disrupting SNPs are located in intronic regions, highlighting the important roles of intronic regions in regulating PD risk genes.

Our study has several strengths. First, considering the high degree of tissue specificity of genetic regulatory elements [75, 76], only ChIP-Seq data from brain tissues or neuronal cell lines were included in this study. This strict criterion guaranteed that only risk variants located in active regulatory regions (with corresponding transcription factors binding) in the brain were examined. Second, we conducted a relatively high-throughput study to systematically characterize the regulatory mechanisms of all the reported PD risk loci and identified functional variants at more than 25% of these loci. Third, we validated the regulatory effects of the 15 identified TF binding-disrupting SNPs with a series of experiments and analyses. Fourth, our study linked the identified TF binding-disrupting SNPs to their potential target genes. Therefore, we have translated the genetic associations into specific genes, an important step for further mechanism dissection and drug development. Finally, we illustrated how the identified functional SNPs conferred the risk for PD by regulating gene expression. For example, our reporter gene assays showed that cells transcribed with different alleles of rs6781790 exhibited significant differences in reporter gene activity, and the C allele led to lower luciferase activity (Fig. 4). Through eQTL analysis, we found that rs6781790 is associated with the expression of several genes in human brain, including *GPX1*, *P4HTM*, *WDR6*, *NCKIPSD*, *AMT*, *CCDC71*, and *DALRD3* (Additional file 1, Figure S3). In addition, GTEx eQTL analysis showed that there were significant associations between PD functional variants and gene expression in the Sustantia Nigra (a key brain region for PD pathogenesis), including the association between rs6781790 and *WDR6* (*P* = 1.6 × 10^−6^) expression. For *AMT* and *DALRD3*, the results of eQTL analysis and reporter gene assays were consistent (i.e., the C allele was associated with lower reporter gene activity and expression of *AMT* and *DALRD3*), suggesting this SNP may contribute to PD risk by regulating the expression of *AMT* and *DALRD3*. We further performed differential expression analysis and found that the expression of *AMT* (*P* = 2.13 × 10^−3^) and *DALRD3* (*P* = 2.93 × 10^−3^) were significantly downregulated in brains of PD cases compared with controls. Taken together, we present convergent and consistent lines of evidence suggesting that rs6781790 may confer PD risk by regulating the expression of *AMT* and *DALRD3.* Therefore, perturbation of the expression of PD risk genes (e.g., *AMT* and *DALRD3*) may underlie the identified functional PD risk variants and have pivotal roles in its pathogenesis.

Single-cell expression analysis of the potential target genes (Table 1) of the identified TF binding-disrupting SNPs showed widespread expression of *GPX1* in many neuronal cell types. However, none of these genes showed cell-specific expression [69] (Additional file 1, Figure S4-S14), suggesting that these genes may have roles in many cell types.

Our study suggests that rs11575895 may be one of the plausible functional SNPs at the 17q21.31 locus. First, Our study has shown that most of the TF binding-disrupting SNPs identified by functional genomics are functional, which is consistent with the findings of previous studies [52–54]. Second, rs11575895 affects the binding of CTCF, RAD21, and SMC3 TFs, and ChIP-Seq data demonstrated that CTCF, RAD21, and SMC3 can bind to the genomic sequence containing rs11575895. Third, reporter gene assays showed that the vector containing G allele of rs11575895 exhibited significantly higher luciferase activity compared with A allele in both SH-SY5Y and U251 cells. Finally, knockdown of CTCF, RAD21, and SMC3 resulted in significant changes in some eQTL genes of rs11575895. These results suggested that rs11575895 may be a functional variant with regulatory effect. However, we noted that rs11575895 is located in the promoter region (or in the first exon, as *MAPT* has several transcripts with different lengths) of *MAPT* (Fig. 5e), a gene that was reported to be associated with PD in previous studies [14–16, 71–74]. *MAPT* encodes the microtubule-associated protein tau (*MAPT*), which promotes microtubule assembly and stability [77] and was associated with frontotemporal dementia [78]. *MAPT* is divided into two major haplotypes, H1 and H2 [79]. Previous studies have shown that H1 haplotype of the *MAPT* is associated with the pathogenesis of PD [80], and a higher H1 expression level was associated with an increased risk of PD [81]. In addition, dysmethylation of *MAPT* promoter was found in leukocytes and brain tissues of PD patients [82, 83]. Though these lines of evidence suggest the functionality of rs11575895, considering the high degree of complexity of this region in PD, more work is needed to validate if rs11575895 is a bona fide functional SNP at this locus.

There are several limitations of this study. First, considering that the main cell types involved in PD pathogenesis are dopaminergic neurons, astrocytes, and microglia, it is ideal to investigate the regulatory effects of risk variants in these cell types. Nevertheless, there are no ChIP-Seq data of dopaminergic neurons and microglia in ENCODE at present. Thus, we only used cell types (including astrocytes) included in ENCODE in this study. We will perform additional analysis once related ChIP-Seq are available, which will provide novel insights into PD pathophysiology. Second, only ChIP-Seq data of 30 TFs were included in this study. Given that there are more than 30 TFs expressed in the brain, risk variants that disrupt TFs not covered in this study might also exert functional impacts on PD. Third, while we have identified TF binding-disrupting SNPs in 11 of the 44 PD risk loci, utilizing only data of the 30 TFs might have limited our identification of such SNPs at the other 33 loci. Finally, only single-nucleotide polymorphisms were analyzed in this study. Considering the importance of other types of genetic variations (e.g., copy number variations (CNVs), chromosomal structural variants, rare mutations, and de novo mutations) in complex disease, further studies are needed to elucidate the genetic mechanisms of PD relevant to these variations.

## Conclusions

In summary, we identified 44 SNPs (from 11 risk loci) affecting the binding of 12 TFs and performed a series of experiments and analyses to validate their regulatory effects. Our study revealed the complex gene regulatory mechanisms of PD risk variants, including widespread disruption of CTCF and POLR2A binding. In addition, our study also pinpoints promising candidate genes for further functional characterization and drug development.

## Supplementary Information

**Additional file 1: Figure S1.** Reporter gene assays validated the regulatory effect of the identified TF binding-disrupting SNPs. **Figure S2.** CTCF, RAD21, and SMC3 knockdown resulted in significant changes of *CRHR1-IT1*, *DND1P1*, *LRRC37A4P*, *MAPT* expression in SH-SY5Y cell lines, indicating that these genes are regulated by the CTCF, RAD21 and SMC3 TFs. **Figure S3.** Boxplots of the eQTL analyses in the LIBD and CMC brain eQTL datasets. **Figure S4.** *AMT* gene expression in single-cell dataset of developing human neocortex (http://solo.bmap.ucla.edu/shiny/webapp/). **Figure S5.** *ARL17A* gene expression in single-cell dataset of developing human neocortex (http://solo.bmap.ucla.edu/shiny/webapp/). **Figure S6.** *DALRD3* gene expression in single-cell dataset of developing human neocortex (http://solo.bmap.ucla.edu/shiny/webapp/). **Figure S7.** *GPX1* gene expression in single-cell dataset of developing human neocortex (http://solo.bmap.ucla.edu/shiny/webapp/). **Figure S8.** *KAT8* gene expression in single-cell dataset of developing human neocortex (http://solo.bmap.ucla.edu/shiny/webapp/). **Figure S9.** *NCKIPSD* gene expression in single-cell dataset of developing human neocortex (http://solo.bmap.ucla.edu/shiny/webapp/). **Figure S10.** *NUPL2* gene expression in single-cell dataset of developing human neocortex (http://solo.bmap.ucla.edu/shiny/webapp/). **Figure S11.** *P4HTM* gene expression in single-cell dataset of developing human neocortex (http://solo.bmap.ucla.edu/shiny/webapp/). **Figure S12.** *PDLIM2* gene expression in single-cell dataset of developing human neocortex (http://solo.bmap.ucla.edu/shiny/webapp/). **Figure S13.** *STX4* gene expression in single-cell dataset of developing human neocortex (http://solo.bmap.ucla.edu/shiny/webapp/). **Figure S14.** *WDR6* gene expression in single-cell dataset of developing human neocortex (http://solo.bmap.ucla.edu/shiny/webapp/). **Table S1.** 44 PD index SNPs used in this study. **Table S2.** PCR primers used to construct DNA fragments for reporter gene assays. **Table S3:** shRNAs used to knockdown of TFs. **Table S4:** RT-qPCR primers used in this study. **Table S6.** Identification of 44 TF binding-disrupting SNPs from the 44 PD risk loci. **Table S7.** 15 TF binding-disrupting SNPs for reporter gene assays. **Table S8.** Summary of the reporter gene assays. **Table S14.** Identification of differentially expressed genes in the prefrontal cortex of PD patients using RNA-Seq.

**Additional file 2: Table S5.** PD index SNPs and SNPs that were in linkage disequilibrium with the index SNPs (r^2^ > 0.6). **Table S9.** Summary of ASE analysis. **Table S10.** PD ASE SNPs were in linkage disequilibrium with coding SNPs. **Table S11.** Association significance between the TF binding-disrupting SNPs and gene expression in the human brain tissues. **Table S12.** Association significance between the TF binding-disrupting SNPs and gene expression in the human brain tissues (at least two brain eQTL datasets). **Table S13.** Association significance between the TF binding-disrupting SNPs and gene expression in the human brain tissues (at least three brain eQTL datasets).

## Data Availability

The PWM data, ChIP-Seq, DNase-Seq, and histone modification of the 44 TF binding-disrupting SNPs are available at SZDB (www.szdb.org) [84, 85].

## Abbreviations

ASE: Allele-specific expression
ChIP-Seq: Chromatin immunoprecipitation sequencing
CMC: Common mind consortium
CNV: Copy number variation
CoDex: Cortical development expression;
DLPFC: Dorsolateral prefrontal cortex
eQTL: Expression quantitative trait loci
FIMO: Find individual motif occurrences
GTEx: Genotype-tissue expression
GWASs: Genome-wide association studies
GWS: Genome-wide significant
HEK293T: Human embryonic kidney cell line
KO: Knockout
LD: Linkage disequilibrium
LIBD: Lieber institute for brain development
LLR: Log-likelihood ratio
PD: Parkinson’s disease
PWM: Position weight matrix
RNA-Seq: RNA sequencing
RT-qPCR: Real-time quantitative PCR
shRNAs: Short hairpin RNAs
SH-SY5Y: Human neuroblastoma cell line
SNPs: Single-nucleotide polymorphisms
TF: Transcription factor
U251: Human glioblastoma cell line
xQTL: xQTL map of the human brains
