# Supplementary material for: Functional genomics elucidates regulatory mechanisms of Parkinson’s disease-associated variants
Source: BMC Med. 2022 Feb 16;20:68. doi: 10.1186/s12916-022-02264-w (PMC8848643; doi:10.1186/s12916-022-02264-w)
Supplement: Supplementary file 1 — Additional file 1: Figure S1. Reporter gene assays validated the regulatory effect of the identified TF binding-disrupting SNPs. Figure S2. CTCF, RAD21, and SMC3 knockdown resulted in significant changes of CRHR1-IT1, DND1P1, LRRC37A4P, MAPT expression in SH-SY5Y cell lines, indicating that these genes are regulated by the CTCF, RAD21 and SMC3 TFs. Figure S3. Boxplots of the eQTL analyses in the LIBD and CMC brain eQTL datasets. Figure S4. AMT gene expression in single-cell dataset of developing human neocortex (http://solo.bmap.ucla.edu/shiny/webapp/). Figure S5. ARL17A gene expression in single-cell dataset of developing human neocortex (http://solo.bmap.ucla.edu/shiny/webapp/). Figure S6. DALRD3 gene expression in single-cell dataset of developing human neocortex (http://solo.bmap.ucla.edu/shiny/webapp/). Figure S7. GPX1 gene expression in single-cell dataset of developing human neocortex (http://solo.bmap.ucla.edu/shiny/webapp/). Figure S8. KAT8 gene expression in single-cell dataset of developing human neocortex (http://solo.bmap.ucla.edu/shiny/webapp/). Figure S9. NCKIPSD gene expression in single-cell dataset of developing human neocortex (http://solo.bmap.ucla.edu/shiny/webapp/). Figure S10. NUPL2 gene expression in single-cell dataset of developing human neocortex (http://solo.bmap.ucla.edu/shiny/webapp/). Figure S11. P4HTM gene expression in single-cell dataset of developing human neocortex (http://solo.bmap.ucla.edu/shiny/webapp/). Figure S12. PDLIM2 gene expression in single-cell dataset of developing human neocortex (http://solo.bmap.ucla.edu/shiny/webapp/). Figure S13. STX4 gene expression in single-cell dataset of developing human neocortex (http://solo.bmap.ucla.edu/shiny/webapp/). Figure S14. WDR6 gene expression in single-cell dataset of developing human neocortex (http://solo.bmap.ucla.edu/shiny/webapp/). Table S1. 44 PD index SNPs used in this study. Table S2. PCR primers used to construct DNA fragments for reporter gene assays. Table S3: shRNAs [file 12916_2022_2264_MOESM1_ESM.doc]

**Supplementary Material for**

**Functional genomics elucidates regulatory mechanisms of Parkinson's disease-associated variants**

Rui Chen^1,2,5^, Jiewei Liu^1,5^, Shiwu Li^1,2^, Xiaoyan Li^1,2^, Yongxia Huo^1^, Yong-Gang Yao^1,2,3^, Xiao Xiao^1^, Ming Li^1,2,3^, Xiong-Jian Luo^1,2,3,4,*^

**Supplementary information**

**Additional file 1, Figure S1:** Reporter gene assays validated the regulatory effect of the identified TF binding-disrupting SNPs.

**Additional file 1, Figure S2:** CTCF, RAD21, and SMC3 knockdown resulted in significant changes of *CRHR1-IT1, DND1P1, LRRC37A4P, MAPT* expression in SH-SY5Y cells, indicating that these genes are regulated by the CTCF, RAD21 and SMC3 TFs.

**Additional file 1, Figure S3:** Boxplots of the eQTL analyses in the LIBD and CMC brain eQTL datasets.

**Additional file 1, Figure S4:** *AMT* gene expression in single-cell dataset of developing human neocortex (<http://solo.bmap.ucla.edu/shiny/webapp/>)

**Additional file 1, Figure S5:** *ARL17A* gene expression in single-cell dataset of developing human neocortex (<http://solo.bmap.ucla.edu/shiny/webapp/>)

**Additional file 1, Figure S6:** *DALRD3* gene expression in single-cell dataset of developing human neocortex (<http://solo.bmap.ucla.edu/shiny/webapp/>)

**Additional file 1, Figure S7:** *GPX1* gene expression in single-cell dataset of developing human neocortex (<http://solo.bmap.ucla.edu/shiny/webapp/>)

**Additional file 1, Figure S8:** *KAT8* gene expression in single-cell dataset of developing human neocortex (<http://solo.bmap.ucla.edu/shiny/webapp/>)

**Additional file 1, Figure S9:** *NCKIPSD* gene expression in single-cell dataset of developing human neocortex (<http://solo.bmap.ucla.edu/shiny/webapp/>)

**Additional file 1, Figure S10:** *NUPL2* gene expression in single-cell dataset of developing human neocortex (<http://solo.bmap.ucla.edu/shiny/webapp/>)

**Additional file 1, Figure S11:** *P4HTM* gene expression in single-cell dataset of developing human neocortex (<http://solo.bmap.ucla.edu/shiny/webapp/>)

**Additional file 1, Figure S12:** *PDLIM2* gene expression in single-cell dataset of developing human neocortex (<http://solo.bmap.ucla.edu/shiny/webapp/>)

**Additional file 1, Figure S13:** *STX4* gene expression in single-cell dataset of developing human neocortex (<http://solo.bmap.ucla.edu/shiny/webapp/>)

**Additional file 1, Figure S14:** *WDR6* gene expression in single-cell dataset of developing human neocortex (<http://solo.bmap.ucla.edu/shiny/webapp/>)

**Additional file 1, Table S1:** 44 PD index SNPs used in this study

**Additional file 1, Table S2:** PCR primers used to construct DNA fragments for reporter gene assays

**Additional file 1, Table S3:** shRNAs used to knockdown of TFs

**Additional file 1, Table S4:** RT-qPCR primers used in this study

**Additional file 2, Table S5:** PD index SNPs and SNPs that were in linkage disequilibrium with the index SNPs (r^2^ > 0.6)

**Additional file 1, Table S6:** Identification of 44 TF binding-disrupting SNPs from the 44 PD risk loci

**Additional file 1, Table S7:** 15 TF binding-disrupting SNPs for reporter gene assays

**Additional file 1, Table S8:** Summary of the reporter gene assays

**Additional file 2, Table S9:** Summary of ASE analysis

**Additional file 2, Table S10:** PD ASE SNPs were in linkage disequilibrium with coding SNPs

**Additional file 2, Table S11:** Association significance between the TF binding-disrupting SNPs and gene expression in the human brain tissues

**Additional file 2, Table S12:** Association significance between the TF binding-disrupting SNPs and gene expression in the human brain tissues (at least two brain eQTL datasets)

**Additional file 2, Table S13:** Association significance between the TF binding-disrupting SNPs and gene expression in the human brain tissues (at least three brain eQTL datasets)

**Additional file 1, Table S14:** Identification of differentially expressed genes in the prefrontal cortex of PD patients using RNA-Seq

| **Table S1. 44 PD index SNPs used in this study** | | |
| --- | --- | --- |
| **Index SNPs^a^** | **Chr** | **Position** |
| rs35749011 | 1 | 155135036 |
| rs823118 | 1 | 205723572 |
| rs4653767 | 1 | 226916078 |
| rs10797576 | 1 | 232664611 |
| rs34043159 | 2 | 102413116 |
| rs6430538 | 2 | 135539967 |
| rs353116 | 2 | 166133632 |
| rs1474055 | 2 | 169110394 |
| rs4073221 | 3 | 18277488 |
| rs12497850 | 3 | 48748989 |
| rs143918452 | 3 | 52816840 |
| rs115185635 | 3 | 87520857 |
| rs12637471 | 3 | 182762437 |
| rs34311866 | 4 | 951947 |
| rs11724635 | 4 | 15737101 |
| rs6812193 | 4 | 77198986 |
| rs356182 | 4 | 90626111 |
| rs78738012 | 4 | 114360372 |
| rs2694528 | 5 | 60273923 |
| rs9468199 | 6 | 27681215 |
| rs9275326 | 6 | 32666660 |
| rs199347 | 7 | 23293746 |
| rs2740594 | 8 | 11707174 |
| rs591323 | 8 | 16697091 |
| rs2280104 | 8 | 22525980 |
| rs13294100 | 9 | 17579690 |
| rs10906923 | 10 | 15569598 |
| rs117896735 | 10 | 121536327 |
| rs3793947 | 11 | 83544472 |
| rs329648 | 11 | 133765367 |
| rs76904798 | 12 | 40614434 |
| rs11060180 | 12 | 123303586 |
| rs11158026 | 14 | 55348869 |
| rs1555399 | 14 | 67984370 |
| rs8005172 | 14 | 88472612 |
| rs2414739 | 15 | 61994134 |
| rs11343 | 16 | 19279464 |
| rs14235 | 16 | 31121793 |
| rs4784227 | 16 | 52599188 |
| rs601999 | 17 | 40698158 |
| rs17649553 | 17 | 43994648 |
| rs12456492 | 18 | 40673380 |
| rs62120679 | 19 | 2363319 |
| rs8118008 | 20 | 3168166 |
| **Note:** ^a^ Index SNPs were from the study of Nalls et al. and Chang et al. | | |

| **Table S2. PCR primers used to construct DNA fragments for reporter gene assays** | |
| --- | --- |
| **Primer name** | **Primer sequence (5'>3')** |
| rs3735901-506-F^a^ | GCCTTCTTTCCTGATGGGATTCT |
| rs3735901-506-R^a^ | GCCACCAAGGGAGGACTGAG |
| rs3735901-C>T^*^ | CAGGTGGGTTGGGGGGCCCCCtGCCGCCCCTCTG |
| rs3735901-C>T^*^ | CAGAGGGGCGGCaGGGGGCCCCCCAACCCACCTG |
| rs6781790-653-F^a^ | CCGTCAAATAAAAAACCACAAGG |
| rs6781790-653-R^a^ | GGCCACATCAGCTTTGTCTCTC |
| rs6781790-T>C^*^ | CTGAGGGcGCTGTTGATGGGCAGCGCGGCGCG |
| rs6781790-T>C^*^ | ATCAACAGCgCCCTCAGCTACAGGTAGCAGAGA |
| rs7599054-544-F^a^ | TCTTTCTTGATGGCACCTTCTGA |
| rs7599054-544-R^a^ | TGGCAATAGCCCAAAGTATAACAA |
| rs7599054-G>A^*^ | ACTAGGGGaCAATATCCATTAATAACAGATCCCTGTC |
| rs7599054-G>A^*^ | GGATATTGtCCCCTAGTGGAAAATTGACAGGA |
| rs11136093-488-F^a^ | GAATGGGGAAGCCCGTCAA |
| rs11136093-488-R^a^ | CCAGGTTGGAGCAGATGGGA |
| rs11136093-C>G^*^ | TCACCTCTGGGCTGGgGGCGGCCCACCCTGCCCC |
| rs11136093-C>G^*^ | CcCCAGCCCAGAGGTGACAGCAGTGTGTTGAG |
| rs11575895-493-F^a^ | CCGCAACGACACAAAGACTCC |
| rs11575895-493-R^a^ | ACGGCGAGGCAGATTTCG |
| rs11575895-A>G^*^ | TgGTGGCCGGAGGAGAAGGCTCCCGCGGAGGC |
| rs11575895-A>G^*^ | TTCTCCTCCGGCCACcAGTGGGCGCGCGCGAGCG |
| rs16833689-575-F^a^ | TGCTCCATCGTCTCCTACCACT |
| rs16833689-575-R^a^ | GAATTGCCAAGACTCTGAGATGAA |
| rs16833689-T>C^*^ | TTTCCTTCTCCTCACcGCCGGGAAGCGGCCATTG |
| rs16833689-T>C^*^ | CgGTGAGGAGAAGGAAAGCTTCCTTGTTCAAG |
| rs17665188-675-F^a^ | GTGGCGTTTCTAATTGCCTTTC |
| rs17665188-675-R^a^ | GGTCTTGCTATATGACCCAAGCTAG |
| rs17665188-T>C^*^ | GATTTGGAcTGCTTACCGCACAGCCTGCTGTA |
| rs17665188-T>C^*^ | GGTAAGCAgTCCAAATCCTAGCAGGAGAATGG |
| rs55787105-505-F^a^ | AATCCTCGTCTCCCAGGTAACAC |
| rs55787105-505-R^a^ | GACCTGAAGTCTAAGTCTGGTGAGC |
| rs55787105-G>A^*^ | AAGCTGGCTCTaAGCCACAGGCCTGGCTGTGA |
| rs55787105-G>A^*^ | TGGCTtAGAGCCAGCTTTGGCATTGCTGCCTG |
| rs62061727-507-F^a^ | GTTTGTTGTCCTTTGGATGCTTCT |
| rs62061727-507-R^a^ | GAAAGTAGTGGGGAGGGGGTAAT |
| rs62061727-G>T | GTCACAGAtCAGGAACAGCCACTCTCCAGTGT |
| rs62061727-G>T^*^ | TGTTCCTGaTCTGTGACTTGGTCCTCGACGCT |
| rs62061809-560-F^a^ | TTTTCAGGGTAAGAGTTAGGGTCAC |
| rs62061809-560-R^a^ | AGCAGTGGCACGATCTCGAC |
| rs62061809-T>C^*^ | ATTTAGTCAGCcGGGCACGGTGGCTCAGGCCT |
| rs62061809-T>C^*^ | TGCCCgGCTGACTAAATTGTTATTCTTATCTAATACATAC |
| rs62064663-747-F^a^ | GGTCGCTGGGAAACATAGAGG |
| rs62064663-747-R^a^ | GCATGGTGGCTCACACCTGTA |
| rs62064663-T>G^*^ | TTTGAAAGCCTgGGGGCGGGGGGTGCAATATT |
| rs62064663-T>G^*^ | GCCCCcAGGCTTTCAAAGCCCCACTGCTCAGG |
| rs117629202-376-F^a^ | TTCTGCAAGCGCGAAATC |
| rs117629202-376-R^a^ | ACTCTGGACACGGTTTATTGC |
| rs117629202-C>A^*^ | CAGGGCGaCCGACCCATGCGGGCCGTTTCGCT |
| rs117629202-C>A^*^ | ATGGGTCGGtCGCCCTGGGATCTCGCGTGCAG |
| rs143191191-414-F^a^ | CTTCCTCCCGACAGGTATACACA |
| rs143191191-414-R^a^ | GAGAGCAAGCTGGATGGTTCC |
| rs143191191-A>/^*^ | TACCAAGCACCTGATGATAACTTGGCTTCCTG |
| rs143191191-A>/^*^ | CATCAGGTGCTTGGTAATATAAACCAGAGCCC |
| rs145273500-434-F^a^ | CTTCTTCCCAGGATGACAGCA |
| rs145273500-434-R^a^ | TAGCCAACTCTATTCTGCGGTCT |
| rs145273500-T>C^*^ | AGCTGAAGcGTGTCATATCCAAGTCATGTCCTTAAC |
| rs145273500-T>C^*^ | TATGACACgCTTCAGCTCTCGTATTGGCTAGAA |
| rs559943616-405-F^a^ | CCCCTAACTCACCAAGCGGA |
| rs559943616-405-R^a^ | AACGAGGTCAGGAACCCAGAAG |
| rs559943616-G>GGA^*^ | AGGCTGGggaGAACTGGGGGCGCCTGCAGGGC |
| rs559943616-G>GGA^*^ | CCAGTTCcctCCAGCCTCCCAGACCTCGCATC |

**Note:** ^a^Represent primers for cloning, ^*^Represent primers for point mutation.

| **Table S3. shRNAs used to knockdown of TFs** | |
| --- | --- |
| **Primer name** | **Primer sequence(5'>3')** |
| Human-*CTCF*-shRNA1-F | **CCGGGAAAGATGCGCTCTAAGAAAGCTCGAGCTTTCTTAGAGCGCATCTTTCTTTTTG** |
| Human-*CTCF*-shRNA1-R | **AATTCAAAAAGAAAGATGCGCTCTAAGAAAGCTCGAGCTTTCTTAGAGCGCATCTTTC** |
| Human-*SIN3A*-shRNA1-F | **CCGGCCCTGAGTTGTTTAATTGGTTCTCGAGAACCAATTAAACAACTCAGGGTTTTTG** |
| Human-*SIN3A*-shRNA1-R | **AATTCAAAAACCCTGAGTTGTTTAATTGGTTCTCGAGAACCAATTAAACAACTCAGGG** |
| Human-*SMC3*-shRNA1-F | **CCGGGTACTGGTCCTCGTGTTATTTCTCGAGAAATAACACGAGGACCAGTACTTTTTG** |
| Human-*SMC3*-shRNA1-R | **AATTCAAAAAGTACTGGTCCTCGTGTTATTTCTCGAGAAATAACACGAGGACCAGTAC** |
| Human-*RAD21*-shRNA1-F | **CCGGGCCATTACTTTACCTGAAGAACTCGAGTTCTTCAGGTAAAGTAATGGCTTTTTG** |
| Human-*RAD21*-shRNA1-R | **AATTCAAAAAGCCATTACTTTACCTGAAGAACTCGAGTTCTTCAGGTAAAGTAATGGC** |

**Note:** CCGG: Age I restriction site; AATT: EcoR I restriction site; CTCGAG: loop; TTTTTG: T-terminate.

| **Table S4. RT-qPCR primers used in this study** | |
| --- | --- |
| **Primer name** | **Primer sequence(5'>3')** |
| Human-CTCF-qPCR-F | TTGTCATGCTCGGTTTACCCA |
| Human-CTCF-qPCR-R | CAATATAGGAATGCTGCTTTCGC |
| Human-RAD21-qPCR-F | CTGCTCAGCCTTTGTGGAATAAC |
| Human-RAD21-qPCR-R | GGTCCTCTCTAGGAACCTCTGGAT |
| Human-SMC3-qPCR-F | AAAAGAGAAGAGGCAGCAGTCAGA |
| Human-SMC3-qPCR-R | TCAGTTCCCAGTTCTGCTTTCAA |
| Human-SIN3A-qPCR-F | TTCTTGTAAACGATTGGGCTCC |
| Human-SIN3A-qPCR-R | TCCTCAGACCACGAAGGGAAG |
| Human-LRRC37A4P-qPCR-F | TCACAAAACTCGCTCCGCAT |
| Human-LRRC37A4P-qPCR-R | TTTATGAGGCTCTTCGCTGCA |
| Human-CRHR1-IT1-qPCR-F | CATTGGGAAGCTGTACTACGACAA |
| Human-CRHR1-IT1-qPCR-R | GGACGATGTTGAAAAGGAAGATG |
| Human-DND1P1-qPCR-F | CAAGTGTTTGGGCATAGGACCT |
| Human-DND1P1-qPCR-R | GCCGTACAGACACAGCATCCTT |
| Human-GPX1-qPCR-F | TGCGGGGCAAGGTACTACTTA |
| Human-GPX1-qPCR-R | CAAACTGGTTGCACGGGAAG |
| Human-P4HTM-qPCR-F | TGTCGGCTCATCATCCATCTG |
| Human-P4HTM-qPCR-R | GCTGACCTGCATAGTGCTCA |
| Human-WDR6-qPCR-F | TGAAACCTTCCACCATAAGCGAT |
| Human-WDR6-qPCR-R | CATGGTGGTGAGATCCCAGAAA |
| Human-MAPT-qPCR-F | CCAAGTGTGGCTCATTAGGCA |
| Human-MAPT-qPCR-R | CCAATCTTCGACTGGACTCTGT |

| **Table S6. Identification of 44 TF binding-disrupting SNPs from the 44 PD risk loci** | | | | | | |
| --- | --- | --- | --- | --- | --- | --- |
| **Index SNP** | **Chr** | **Index** | **Functional** | **Functional** | **r^2^** | **Disrupt TFs** |
|  |  | **SNP Position** | **Candidates** | **SNP Position** |  |  |
| rs12497850 | 3 | 48748989 | rs6781790 | 49044767 | 0.906402 | SIN3A/REST |
| rs12497850 | 3 | 48748989 | rs9840684 | 48893780 | 0.910411 | CTCF/RAD21 |
| rs12637471 | 3 | 182762437 | rs16833689 | 182803656 | 0.699684 | CTCF/RAD21 |
| rs14235 | 16 | 31121793 | rs8050894 | 31104509 | 0.860353 | RAD21 |
| rs143918452 | 3 | 52816840 | rs145273500 | 52704972 | 1 | PBX3 |
| rs143918452 | 3 | 52816840 | rs141535281 | 52227274 | 1 | TCF12 |
| rs143918452 | 3 | 52816840 | rs140163861 | 52227289 | 1 | NFIC |
| rs143918452 | 3 | 52816840 | rs74735459 | 52252996 | 1 | CTCF |
| rs143918452 | 3 | 52816840 | rs146527642 | 52271779 | 1 | EP300/REST |
| rs1474055 | 2 | 169110394 | rs76179989 | 169103999 | 0.990628 | CTCF |
| rs17649553 | 17 | 43994648 | rs11575895 | 43971785 | 0.994629 | CTCF/RAD21/SMC3 |
| rs17649553 | 17 | 43994648 | rs17665188 | 44357351 | 0.956933 | REST |
| rs17649553 | 17 | 43994648 | rs55787105 | 43853109 | 0.994629 | NFIC |
| rs17649553 | 17 | 43994648 | rs62061727 | 44017124 | 0.994629 | POLR2A |
| rs17649553 | 17 | 43994648 | rs62061809 | 44178839 | 0.989251 | TCF12 |
| rs17649553 | 17 | 43994648 | rs62064663 | 44080039 | 0.989251 | CTCF/SMC3 |
| rs17649553 | 17 | 43994648 | rs111825734 | 44019107 | 0.952478 | POLR2A |
| rs17649553 | 17 | 43994648 | rs117629202 | 44344596 | 0.790129 | CTCF |
| rs17649553 | 17 | 43994648 | rs143191191 | 44341868 | 0.978751 | USF1/TCF12 |
| rs17649553 | 17 | 43994648 | rs559943616 | 43568280 | 0.758665 | POLR2A/CTCF |
| rs17649553 | 17 | 43994648 | rs17688249 | 43766754 | 0.994629 | REST |
| rs17649553 | 17 | 43994648 | rs56046792 | 43797246 | 0.994629 | REST |
| rs17649553 | 17 | 43994648 | rs62054378 | 43799667 | 0.994629 | REST |
| rs17649553 | 17 | 43994648 | rs2864087 | 43807063 | 0.994629 | USF1 |
| rs17649553 | 17 | 43994648 | rs7350923 | 43834970 | 0.994629 | NFIC |
| rs17649553 | 17 | 43994648 | rs12150515 | 44090685 | 0.989251 | POLR2A |
| rs17649553 | 17 | 43994648 | rs62062136 | 44116312 | 0.989251 | POLR2A |
| rs17649553 | 17 | 43994648 | rs876944 | 44134391 | 0.989251 | RXRA |
| rs17649553 | 17 | 43994648 | rs62060792 | 44141955 | 0.989251 | POLR2A |
| rs17649553 | 17 | 43994648 | rs974291 | 44150480 | 0.989251 | SIN3A |
| rs17649553 | 17 | 43994648 | rs1468240 | 44196447 | 0.973245 | POLR2A |
| rs17649553 | 17 | 43994648 | rs199442 | 44820122 | 0.697656 | TCF12 |
| rs17649553 | 17 | 43994648 | rs199536 | 44820425 | 0.689387 | EP300/NFIC |
| rs17649553 | 17 | 43994648 | rs199523 | 44848517 | 0.649932 | CTCF |
| rs199347 | 7 | 23293746 | rs10270788 | 23189000 | 0.629274 | POLR2A |
| rs199347 | 7 | 23293746 | rs858305 | 23242830 | 0.791519 | POLR2A |
| rs2280104 | 8 | 22525980 | rs3735901 | 22462374 | 0.824774 | RAD21 |
| rs2280104 | 8 | 22525980 | rs11136093 | 22479988 | 0.830891 | SIN3A/REST |
| rs2280104 | 8 | 22525980 | rs2272718 | 22457388 | 0.817831 | REST |
| rs2280104 | 8 | 22525980 | rs878051 | 22483836 | 0.832149 | CTCF |
| rs2740594 | 8 | 11707174 | rs1692821 | 11699988 | 0.718 | POLR2A |
| rs2740594 | 8 | 11707174 | rs1736082 | 11703420 | 0.848016 | CTCF/RAD21/SMC3 |
| rs35749011 | 1 | 155135036 | rs12752133 | 155205378 | 0.860057 | POLR2A |
| rs6430538 | 2 | 135539967 | rs7599054 | 135540546 | 1 | CTCF/RAD21/SMC3 |

| **Table S7. 15 TF binding-disrupting SNPs for reporter gene assays** | | | | | | |
| --- | --- | --- | --- | --- | --- | --- |
| **SNP ID** | **Chr** | **Ref^a^** | **Alt^b^** | **Location** | **Nearby Genes^c^** | **Disrupted TFs** |
| rs3735901 | Chr8 | T | C | 22604861 | CCAR2 | RAD21 |
| rs6781790 | Chr3 | C | T | 49007334 | P4HTM | SIN3A/REST |
| rs7599054 | Chr2 | A | G | 134782976 | TMEM163 | CTCF/RAD21/SMC3 |
| rs11136093 | Chr8 | C | G | 22622475 | CCAR2 | SIN3A/REST |
| rs11575895 | Chr17 | A | G | 45894419 | MAPT | CTCF/RAD21/SMC3 |
| rs16833689 | Chr3 | C | T | 183085868 | MCCC1 | CTCF/RAD21 |
| rs17665188 | Chr17 | T | C | 46279985 | ARL17B | REST |
| rs55787105 | Chr17 | G | A | 45775743 | CRHR1 | NFIC |
| rs62061727 | Chr17 | G | T | 45939758 | MAPT | POLR2A |
| rs62061809 | Chr17 | T | C | 46101473 | KANSL1 | TCF12 |
| rs62064663 | Chr17 | T | G | 46002673 | MAPT | CTCF/SMC3 |
| rs117629202 | Chr17 | C | A | 46267230 | ARL17B | CTCF |
| rs143191191 | Chr17 | A | - | 46264503 | ARL17B | USF1/TCF12 |
| rs145273500 | Chr3 | T | C | 52670956 | PBRM1 | PBX3 |
| rs559943616 | Chr17 | G | GAG | 45490915 | PLEKHM1 | POLR2A/CTCF |

**Note:** ^a^ Reference allele, ^b^ Alternative allele. ^c^ Nearby genes of the TF binding-disrupting SNPs.

| **Table S8. Summary of the reporter gene assays** | | |
| --- | --- | --- |
| If allelic differences at the identified regulatory SNPs affect  luciferase activity significantly | | |
| **Regulatory SNP id** | **SH-SY5Y** | **SK-N-SH** |
| rs3735901 | Yes | Yes |
| rs6781790 | Yes | Yes |
| rs7599054 | No | No |
| rs11136093 | Yes | Yes |
| rs11575895 | Yes | Yes |
| rs16833689 | No | No |
| rs17665188 | Yes | Yes |
| rs55787105 | Yes | Yes |
| rs62061727 | Yes | Yes |
| rs62061809 | Yes | Yes |
| rs62064663 | Yes | Yes |
| rs117629202 | No | No |
| rs143191191 | Yes | Yes |
| rs145273500 | No | No |
| rs559943616 | Yes | Yes |

| **Table S14. Identification of differentially expressed genes in the prefrontal cortex of  PD patients using RNA-Seq** | | | | |
| --- | --- | --- | --- | --- |
| **Gene** | **Symbol** | **P value** | **Q value** | **Log FC** |
| ENSG00000145020 | *AMT* | 0.002149556 | 0.039000165 | -0.600839786 |
| ENSG00000178149 | *DALRD3* | 0.002925021 | 0.045190621 | -0.522787873 |
| ENSG00000136235 | *GPNMB* | 0.002216839 | 0.039475393 | 1.123769371 |
| ENSG00000008853 | *RHOBTB2* | 0.000328456 | 0.01550347 | -0.685804226 |
| These data were from the study of Marshall et al. | | | | |


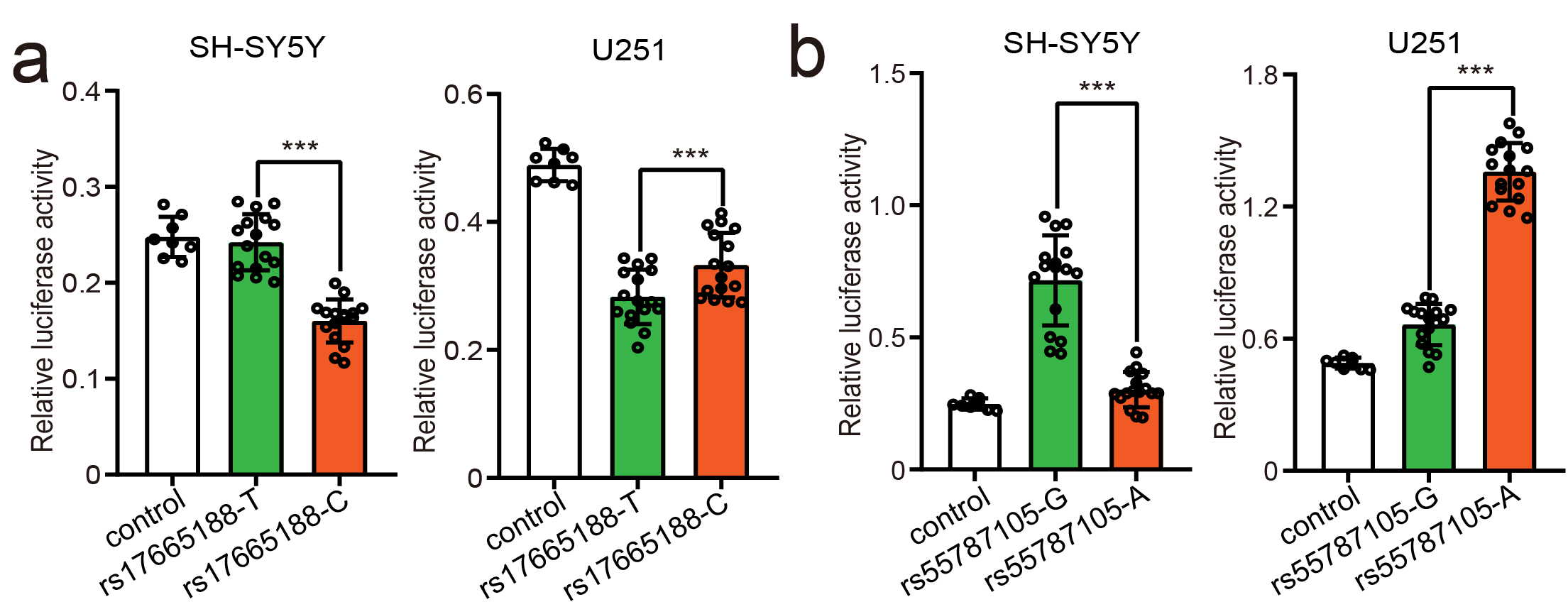


**Figure S1. Reporter gene assays validated the regulatory effect of the identified TF binding-disrupting SNPs.** N = 8 for the control group, n = 16 per experimental group for SH-SY5Y and U251 cells. Two-tailed *Student’s t* test was used for statistical analyses. *P < 0.05, **P < 0.01, ***P < 0.001.

**
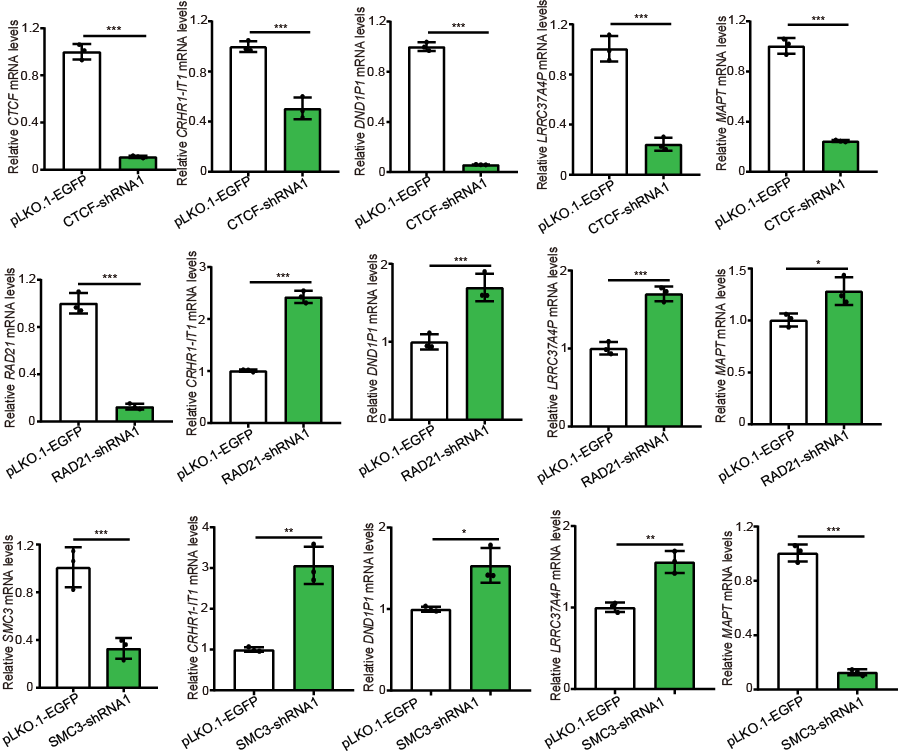
**

**Figure S2. CTCF, RAD21 and SMC3 knockdown resulted in significant changes of *CRHR1-IT1, DND1P1, LRRC37A4P, MAPT* expression in SH-SY5Y cells, indicating that these genes are regulated by the CTCF, RAD21 and SMC3 TFs.** Three replicates from three independent biological samples were used for statistical analysis, and two-tailed *Student’s t test* was used to test whether the difference reaches significance level (0.05).


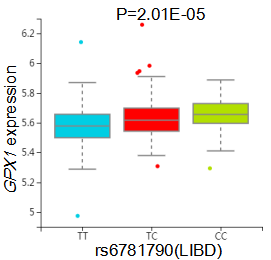

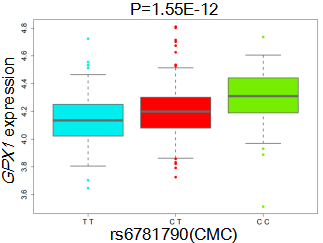

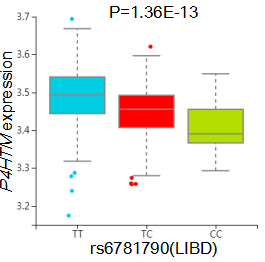

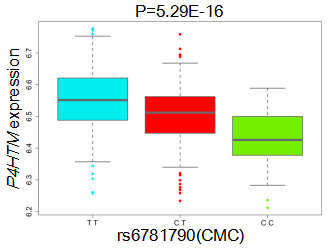


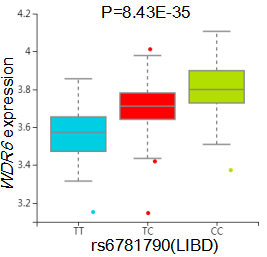

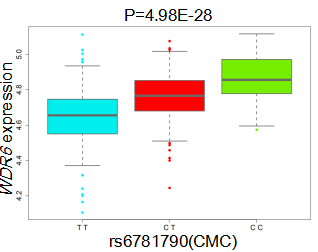

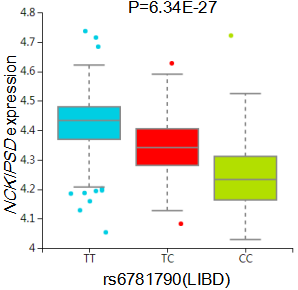

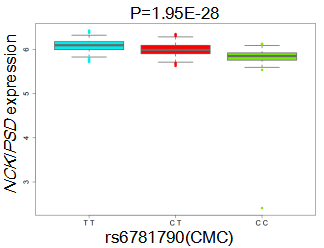

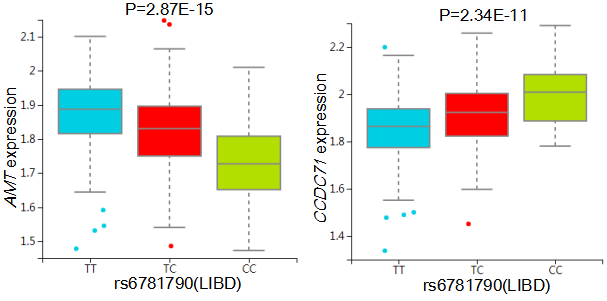


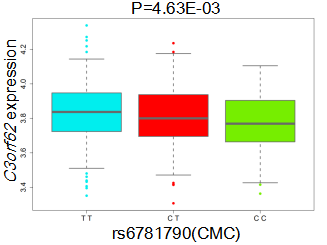

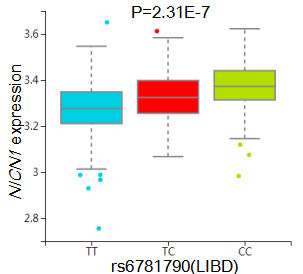

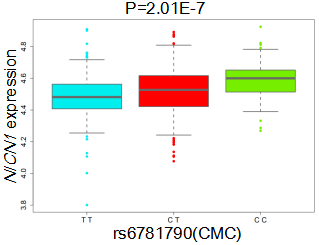

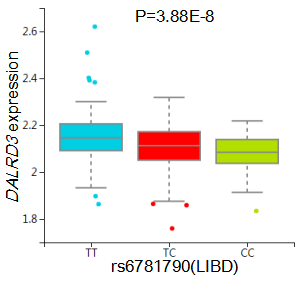

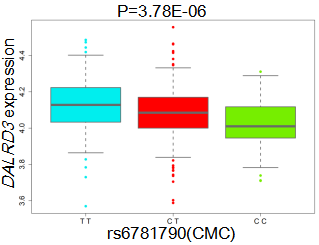


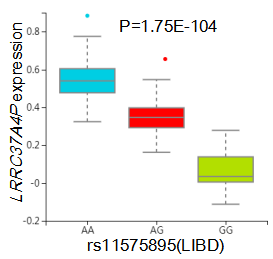

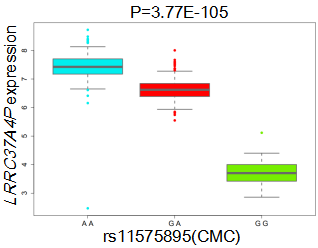

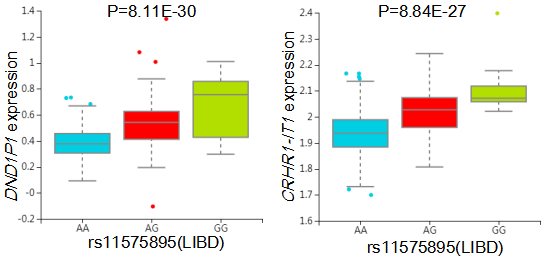

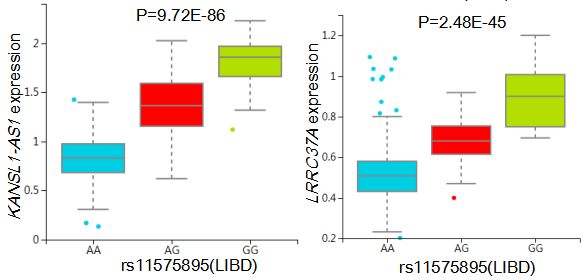


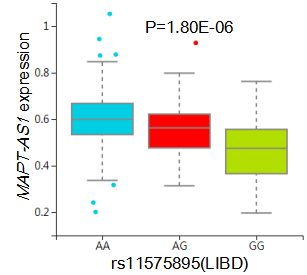

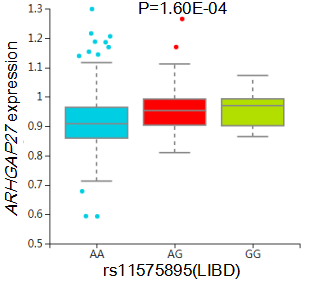

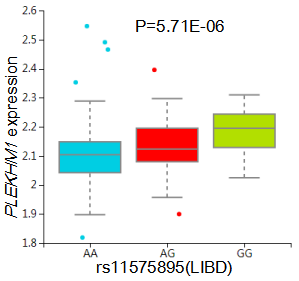

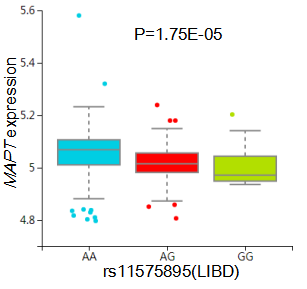


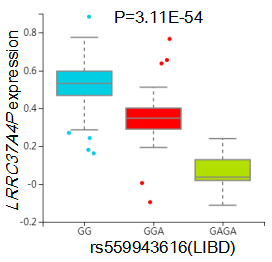

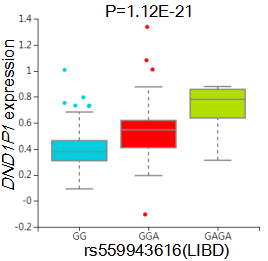


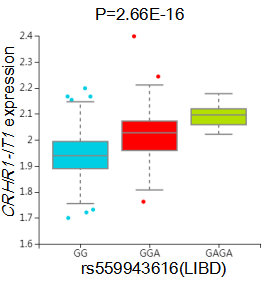

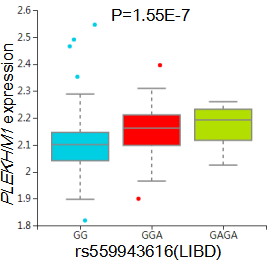


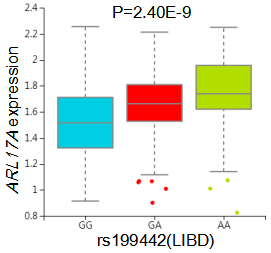

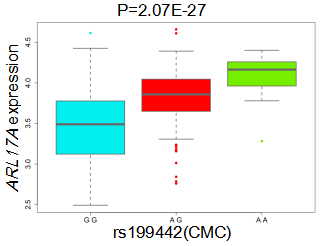


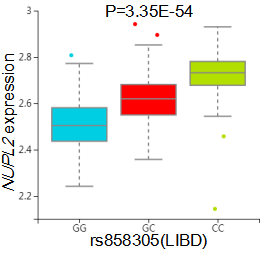

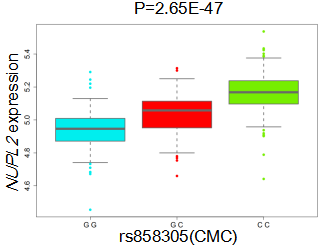


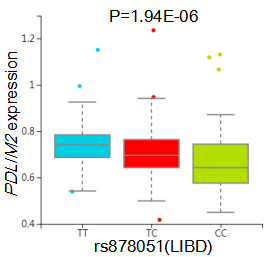

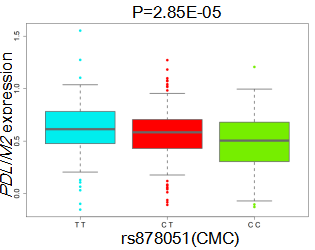

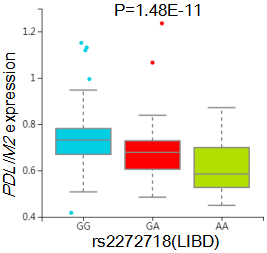

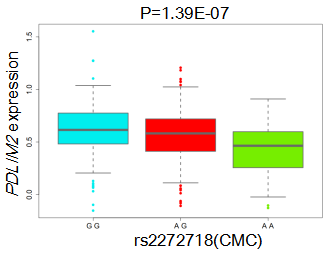

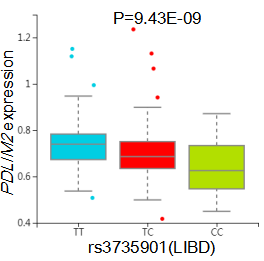

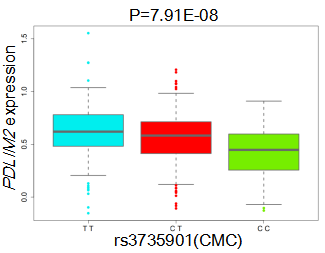


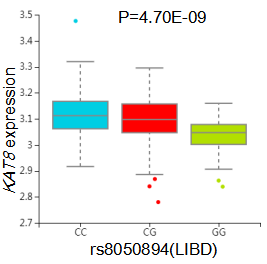

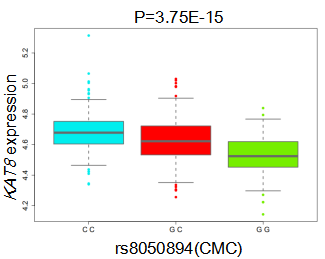

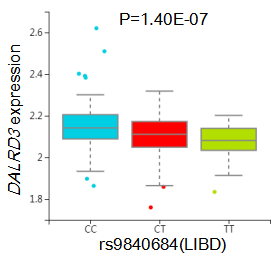

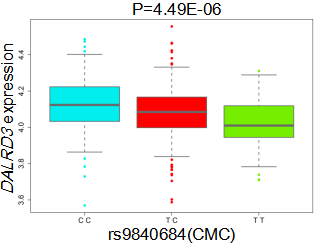

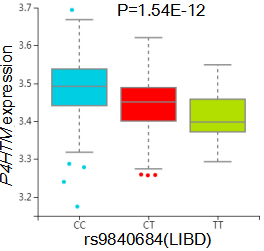

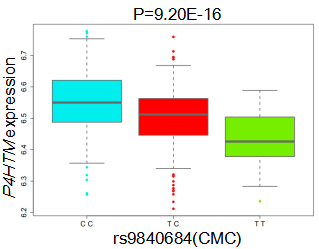


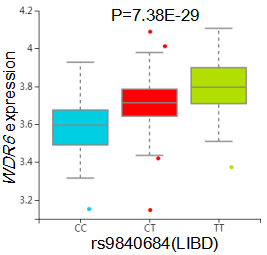

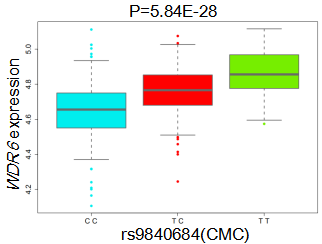

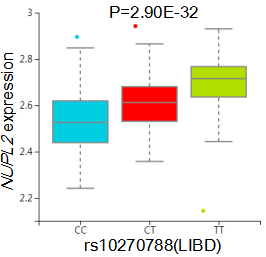

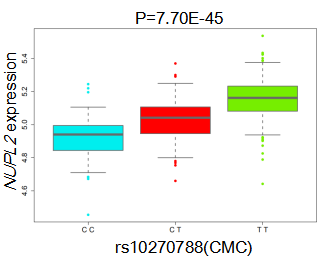

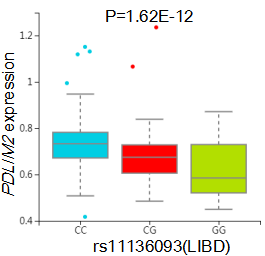

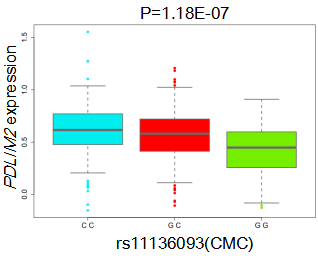


**Figure S3. Boxplots of the eQTL analyses in the LIBD and CMC brain eQTL datasets.** Brain tissues from the Common Mind Consortium (CMC) (N=467), the Lieber Institute for Brain Development (LIBD) brain eQTL (N=412).

**
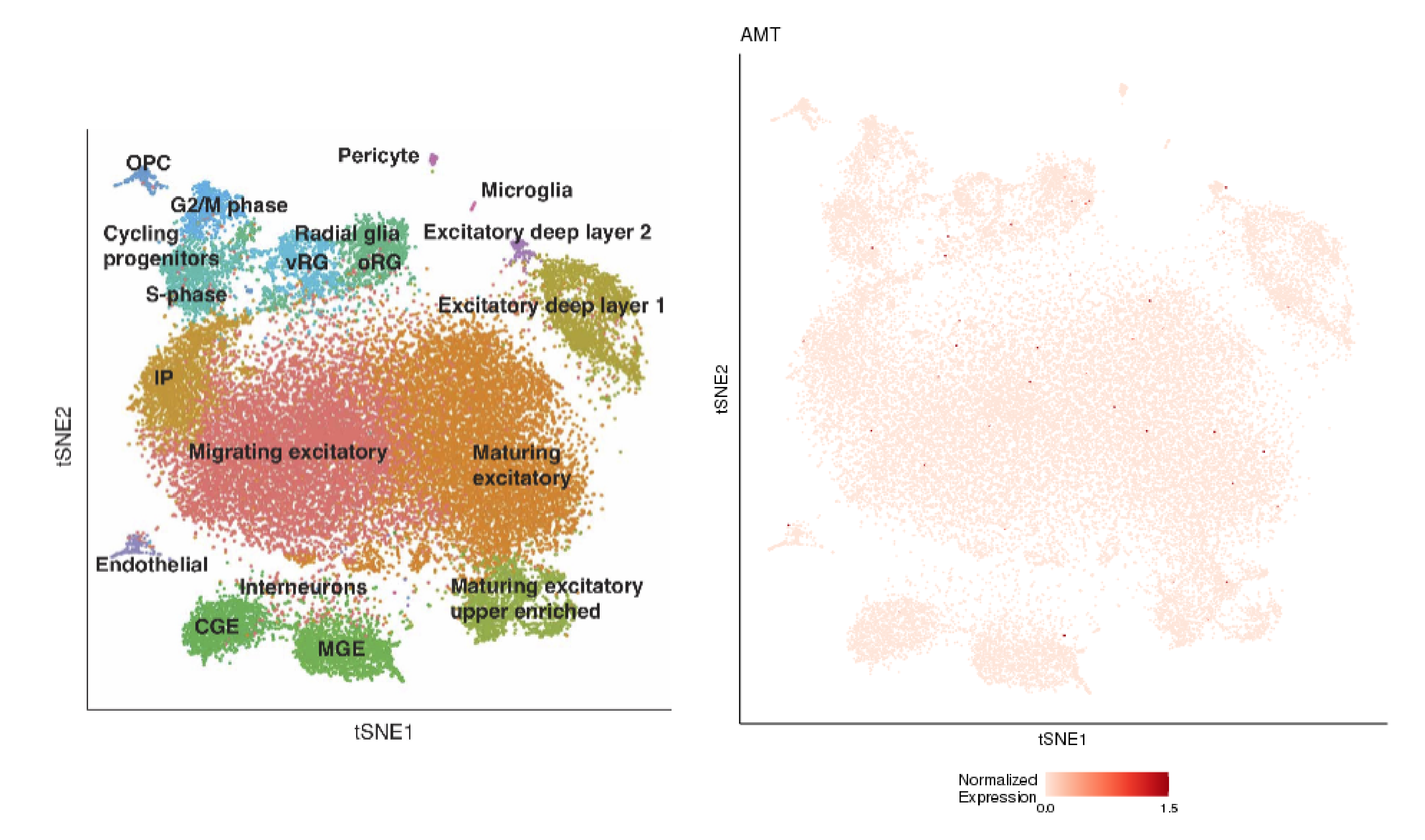
**

**Figure S4. *AMT* gene expression in single-cell dataset of developing human neocortex (**[**http://solo.bmap.ucla.edu/shiny/webapp/**](http://solo.bmap.ucla.edu/shiny/webapp/)**)**

**
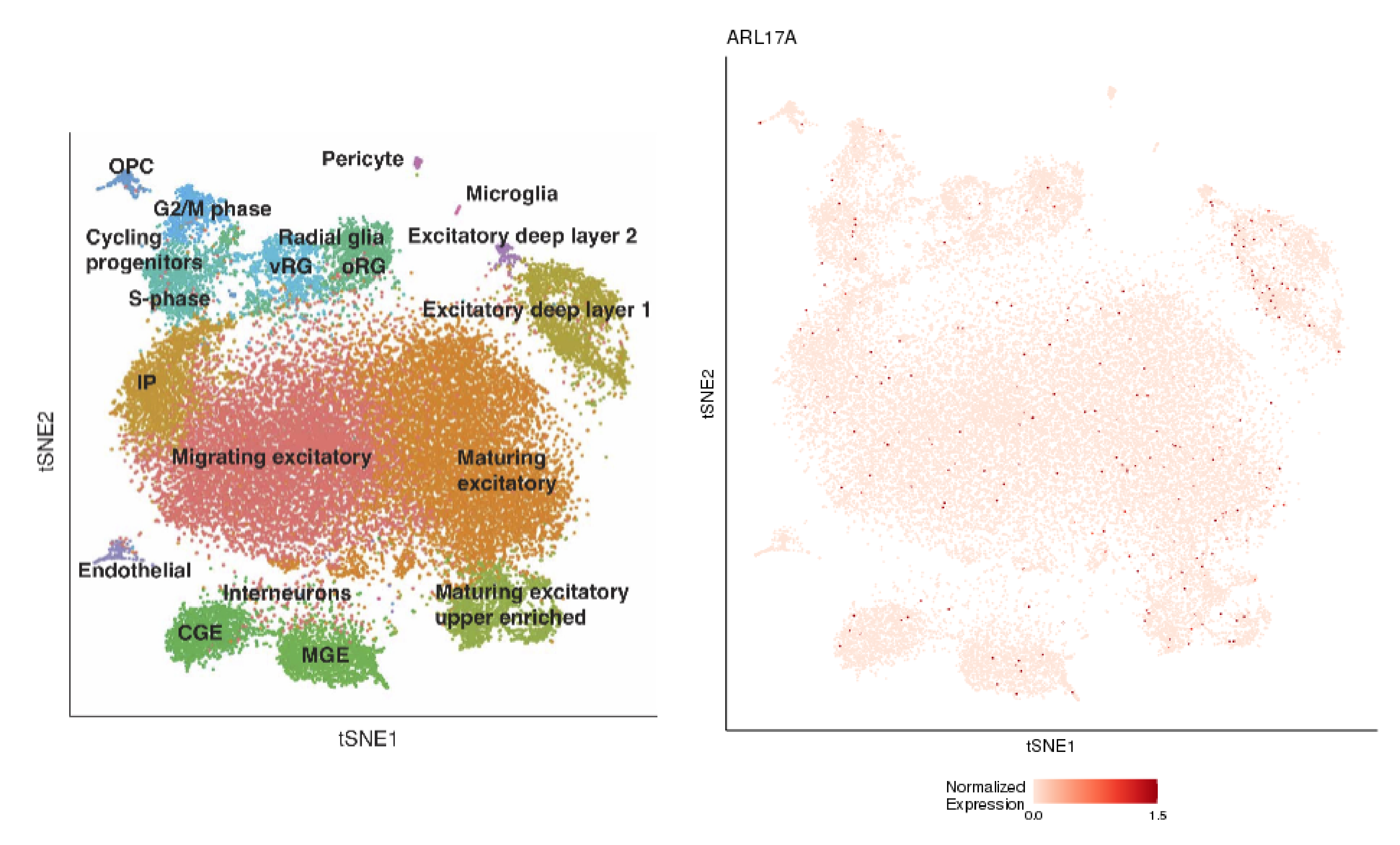
**

**Figure S5. *ARL17A* gene expression in single-cell dataset of developing human neocortex (**[**http://solo.bmap.ucla.edu/shiny/webapp/**](http://solo.bmap.ucla.edu/shiny/webapp/)**)**

**
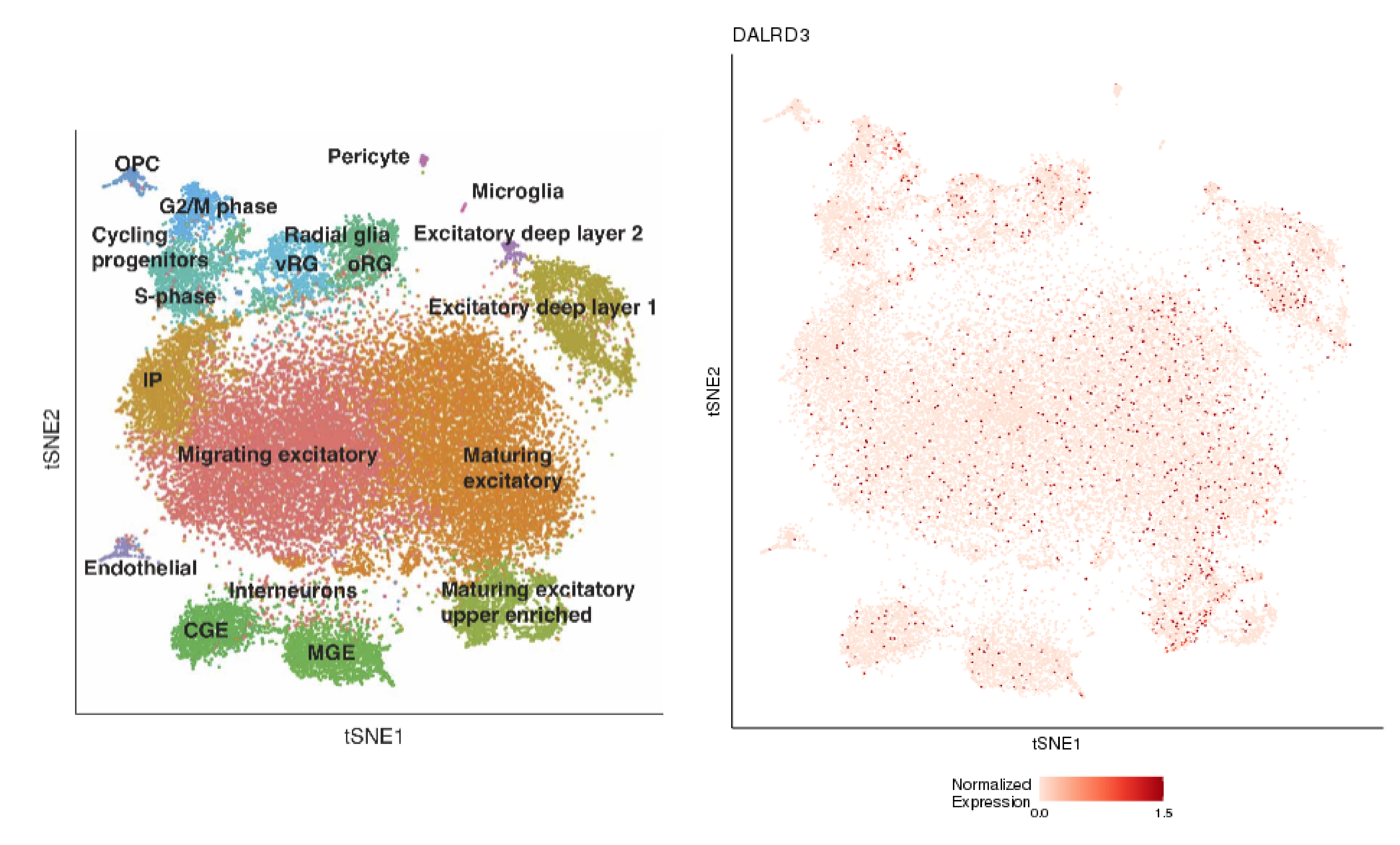
**

**Figure S6. *DALRD3* gene expression in single-cell dataset of developing human neocortex (**[**http://solo.bmap.ucla.edu/shiny/webapp/**](http://solo.bmap.ucla.edu/shiny/webapp/)**)**

**
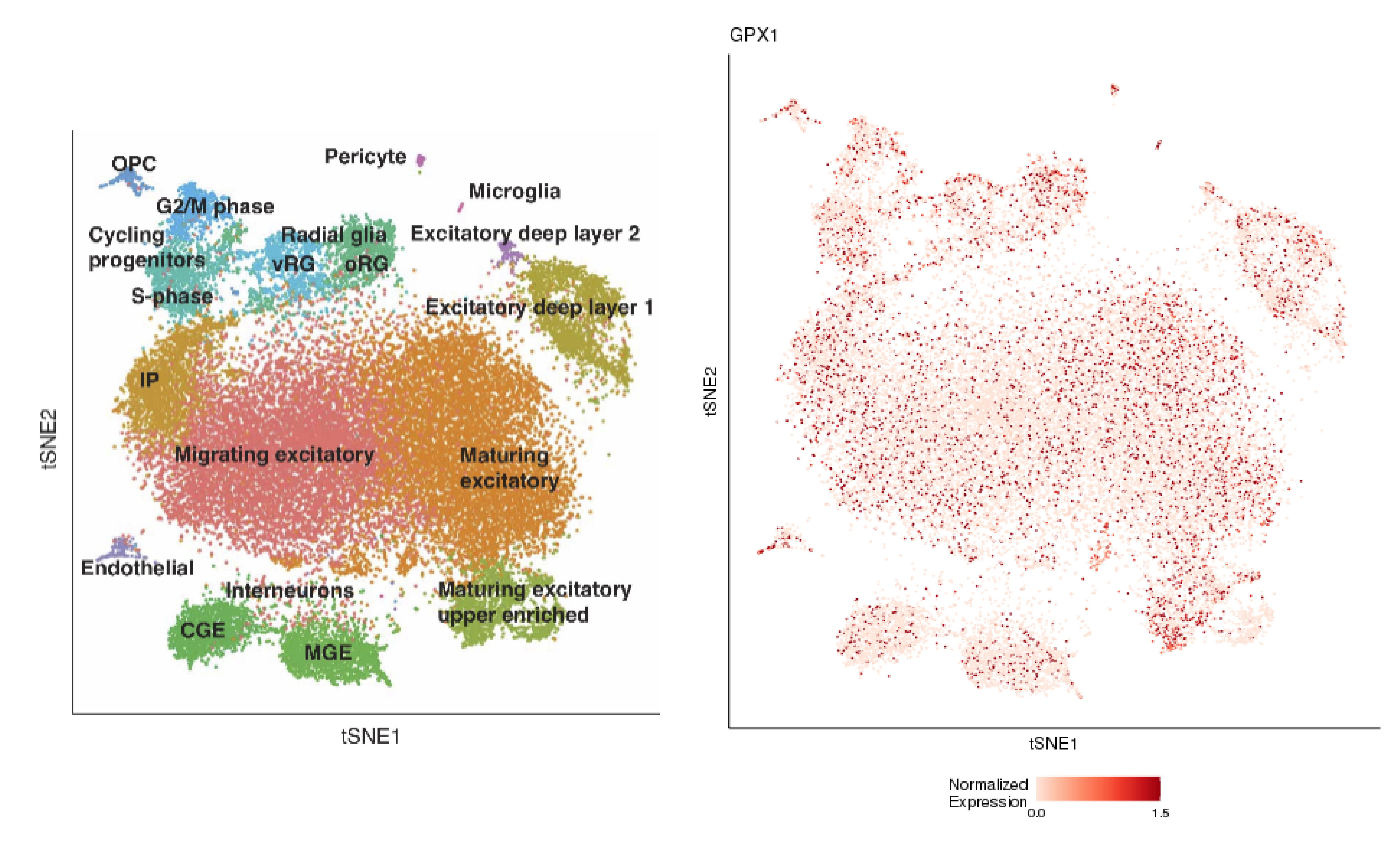
**

**Figure S7. *GPX1* gene expression in single-cell dataset of developing human neocortex (**[**http://solo.bmap.ucla.edu/shiny/webapp/**](http://solo.bmap.ucla.edu/shiny/webapp/)**)**

**
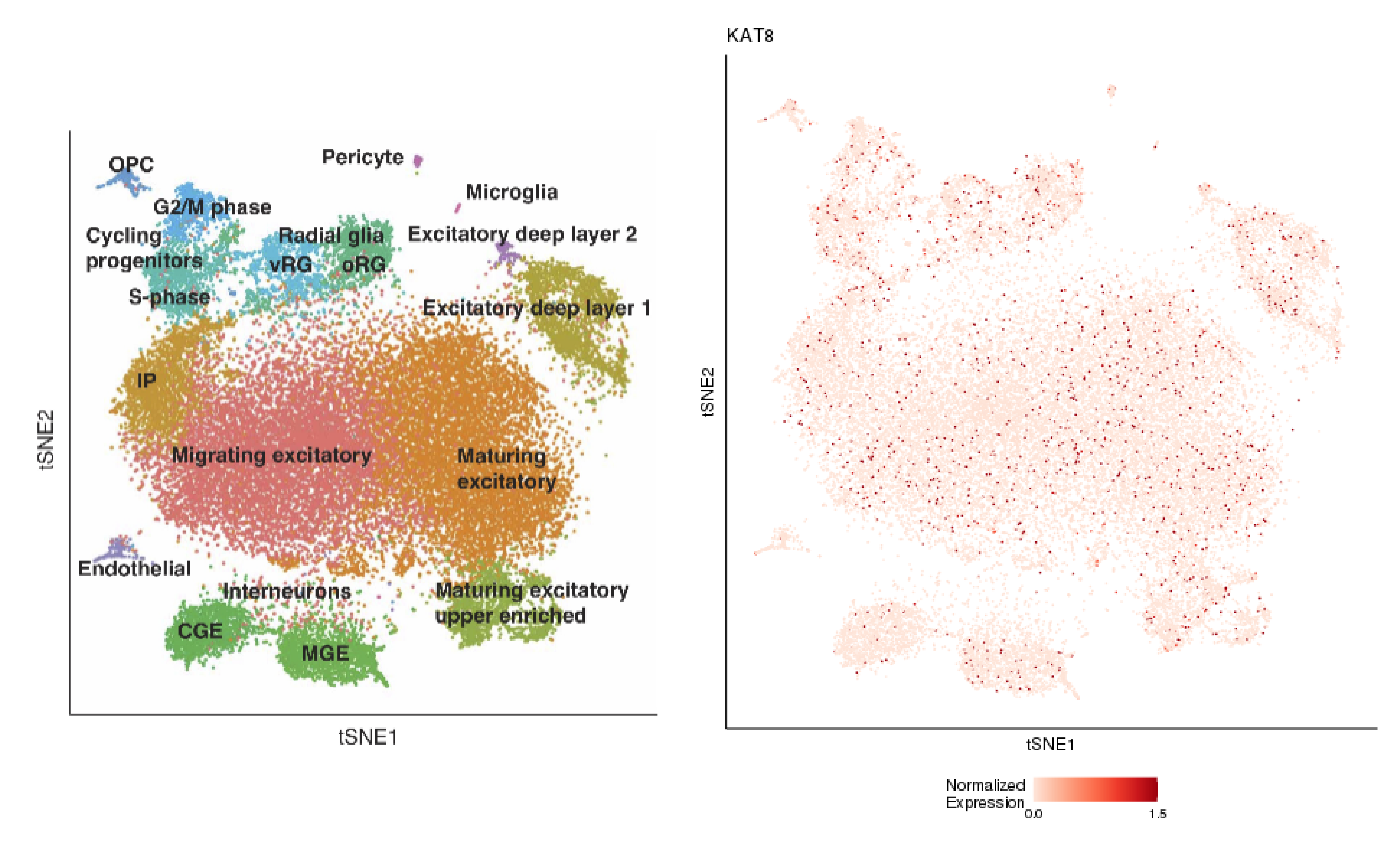
**

**Figure S8. *KAT8* gene expression in single-cell dataset of developing human neocortex (**[**http://solo.bmap.ucla.edu/shiny/webapp/**](http://solo.bmap.ucla.edu/shiny/webapp/)**)**

**
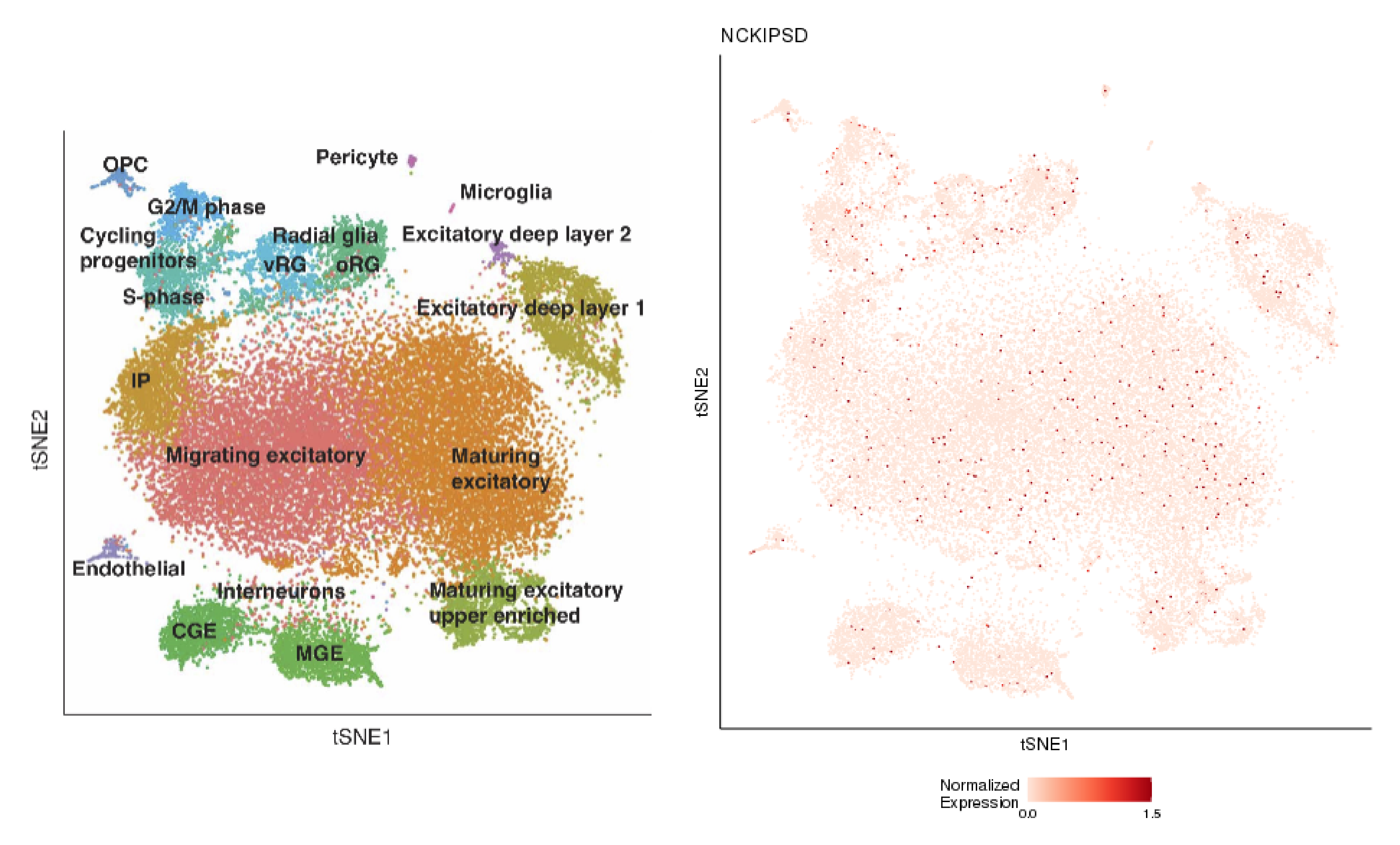
**

**Figure S9. *NCKIPSD* gene expression in single-cell dataset of developing human neocortex (**[**http://solo.bmap.ucla.edu/shiny/webapp/**](http://solo.bmap.ucla.edu/shiny/webapp/)**)**

**
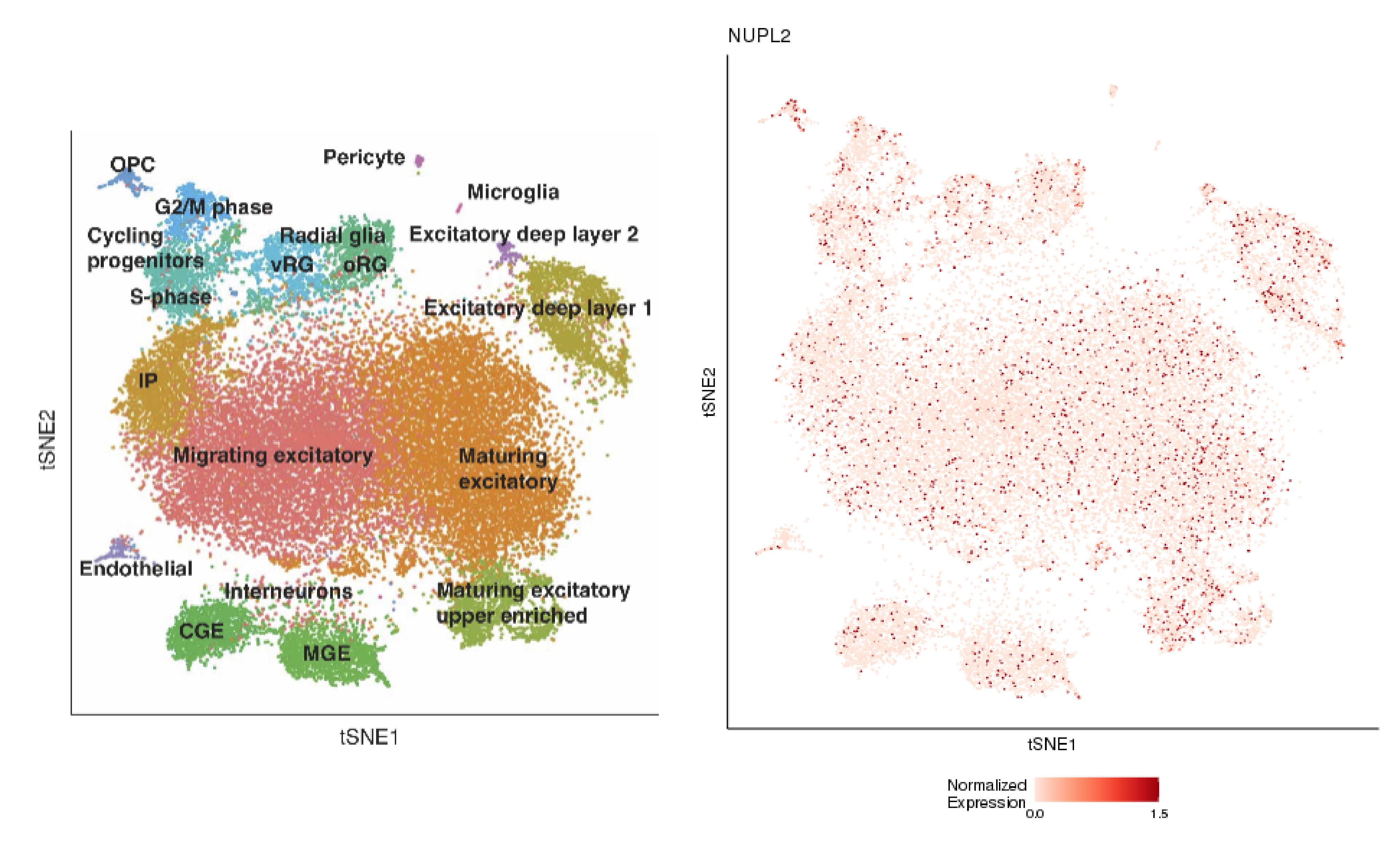
**

**Figure S10. *NUPL2* gene expression in single-cell dataset of developing human neocortex (**[**http://solo.bmap.ucla.edu/shiny/webapp/**](http://solo.bmap.ucla.edu/shiny/webapp/)**)**

**
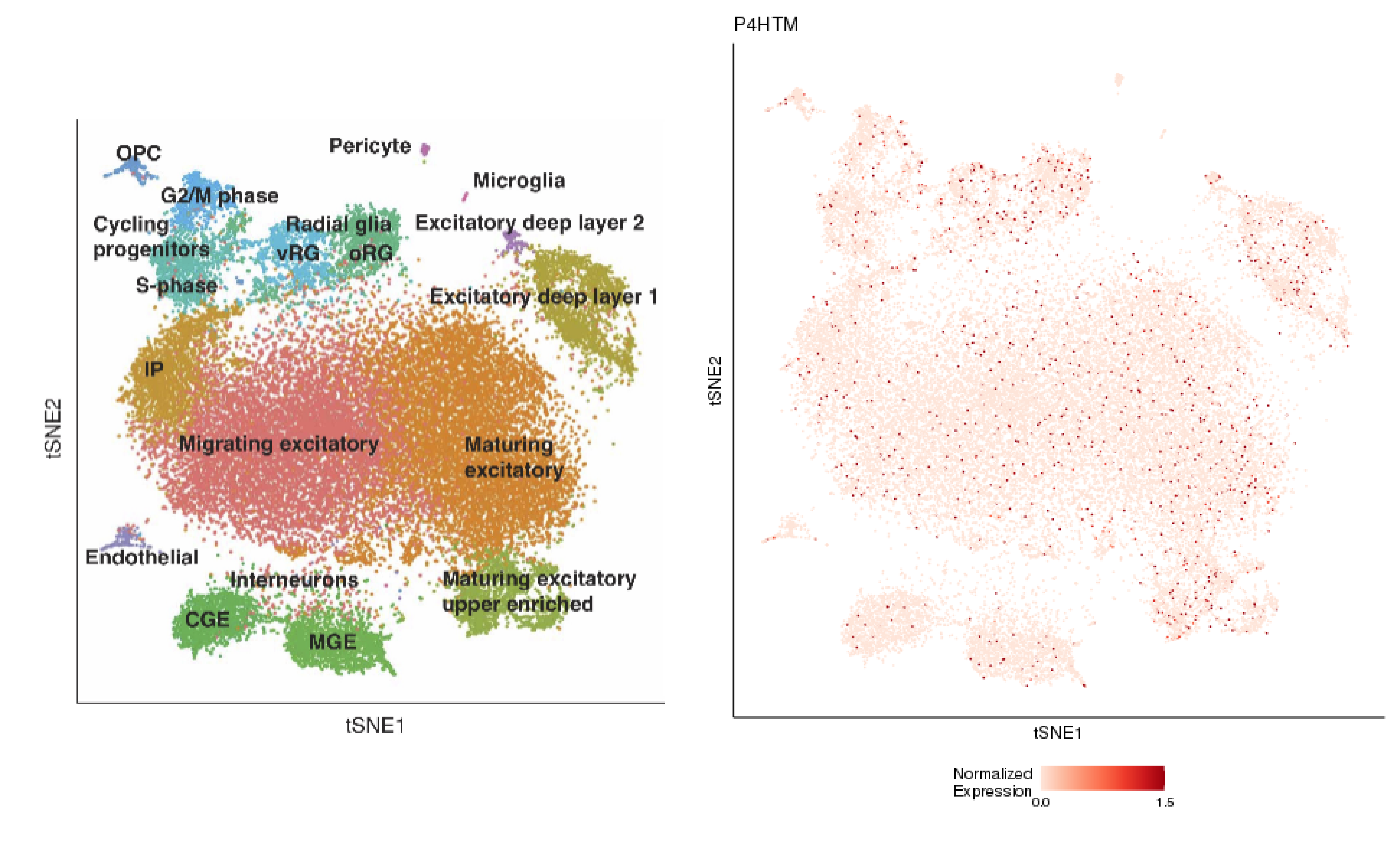
**

**Figure S11. *P4HTM* gene expression in single-cell dataset of developing human neocortex (**[**http://solo.bmap.ucla.edu/shiny/webapp/**](http://solo.bmap.ucla.edu/shiny/webapp/)**)**

**
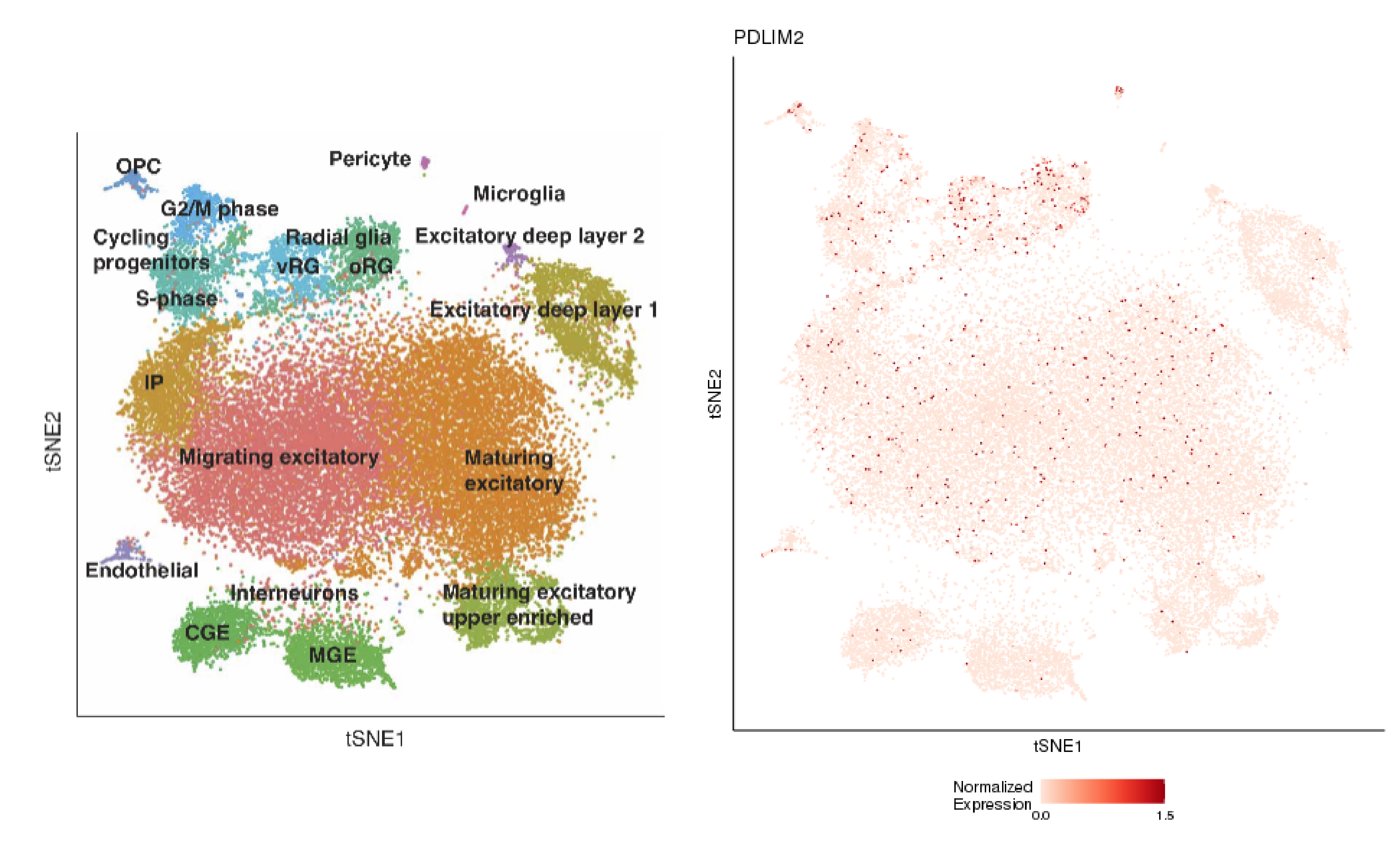
**

**Figure S12. *PDLIM2* gene expression in single-cell dataset of developing human neocortex (**[**http://solo.bmap.ucla.edu/shiny/webapp/**](http://solo.bmap.ucla.edu/shiny/webapp/)**)**

**
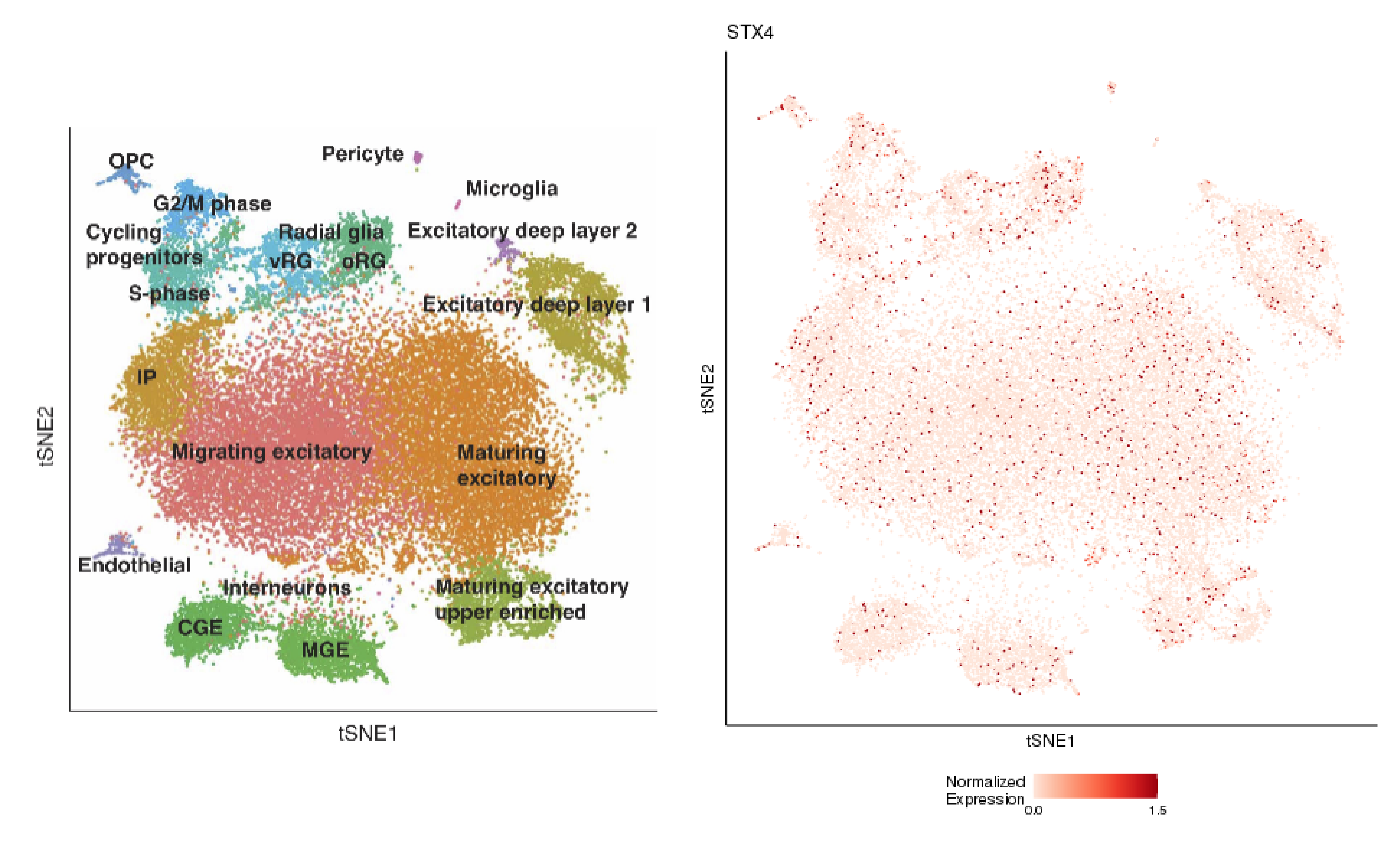
**

**Figure S13. *STX4* gene expression in single-cell dataset of developing human neocortex (**[**http://solo.bmap.ucla.edu/shiny/webapp/**](http://solo.bmap.ucla.edu/shiny/webapp/)**)**

**
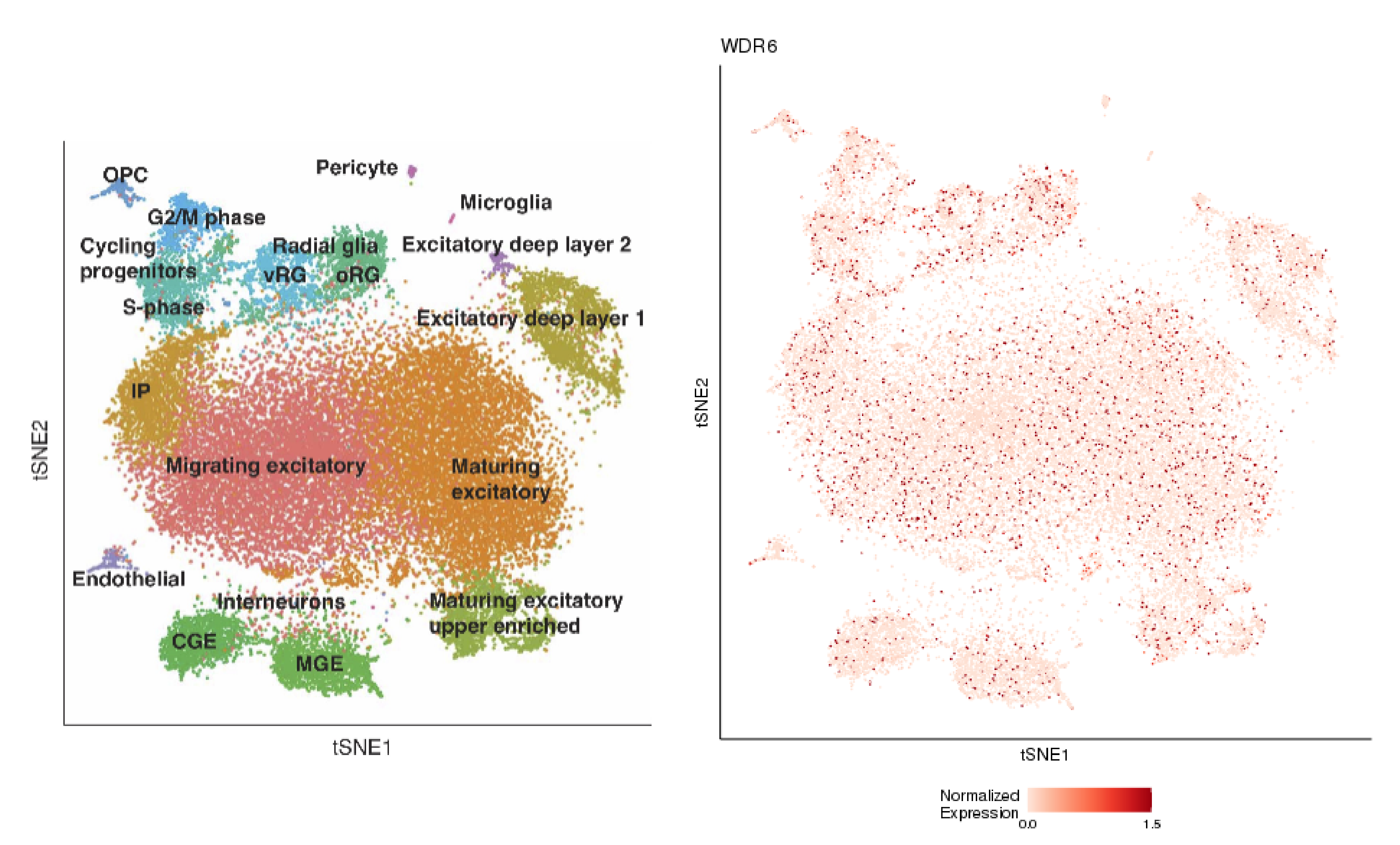
**

**Figure S14. *WDR6* gene expression in single-cell dataset of developing human neocortex (**[**http://solo.bmap.ucla.edu/shiny/webapp/**](http://solo.bmap.ucla.edu/shiny/webapp/)**)**
